# Bacterial processing of glucose modulates *C. elegans* lifespan and healthspan

**DOI:** 10.1038/s41598-021-85046-3

**Published:** 2021-03-15

**Authors:** Samuel F. Kingsley, Yonghak Seo, Calista Allen, Krishna S. Ghanta, Steven Finkel, Heidi A. Tissenbaum

**Affiliations:** 1 Department of Molecular, Cell and Cancer Biology, University of Massachusetts Medical School, Worcester, MA 01605 USA; 2 Molecular and Computational Biology Section, Department of Biological Sciences, University of Southern California, Los Angeles, CA 90089 USA; 3 RNA Therapeutics Institute, University of Massachusetts Medical School, Worcester, MA 01605 USA; 4 Program in Molecular Medicine, University of Massachusetts Medical School, Worcester, MA 01605 USA

**Keywords:** Genetics, Molecular biology

## Abstract

Intestinal microbiota play an essential role in the health of a host organism. Here, we define how commensal *Escherichia coli (E. coli)* alters its host after long term exposure to glucose using a *Caenorhabditis elegans*-*E. coli* system where only the bacteria have direct contact with glucose. Our data reveal that bacterial processing of glucose results in reduced lifespan and healthspan including reduced locomotion, oxidative stress resistance, and heat stress resistance in *C. elegans*. With chronic exposure to glucose, *E. coli* exhibits growth defects and increased advanced glycation end products. These negative effects are abrogated when the *E. coli* is not able to process the additional glucose and by the addition of the anti-glycation compound carnosine. Physiological changes of the host *C. elegans* are accompanied by dysregulation of detoxifying genes including glyoxalase, glutathione-S-transferase, and superoxide dismutase. Loss of the glutathione-S-transferase, *gst-4* shortens *C. elegans* lifespan and blunts the animal's response to a glucose fed bacterial diet. Taken together, we reveal that added dietary sugar may alter intestinal microbial *E. coli* to decrease lifespan and healthspan of the host and define a critical role of detoxification genes in maintaining health during a chronic high-sugar diet.

## Introduction

Approximately 0.1–5% of the human microbiome consists of the bacterium *E. coli*, which is normally thought to present little to no harm to its hosts (reviewed in^1,2^). Despite this small fraction, *E. coli* is responsible for thousands of illnesses each year due to its rapid colonization of the human gut^2,3^. Overall, the microbiota of the intestine plays a major role in human health and disease and undergoes large changes as we age^4,5^. Additionally, recent studies suggest that a high sugar diet can alter the gut microbiota leading to age-associated illness^6–9^. Direct evidence of the overall importance of the human intestinal microbiota particularly with respect to carbohydrate metabolism, and mechanistic analysis has not been possible due to its species complexity, containing hundreds of types of organisms. In addition, there is limited opportunity to perform experiments on humans and as such, human microbiome studies are defined primarily by fecal samples which mainly represent only a small portion of the large intestine microbial community.

*Caenorhabditis elegans* present a highly malleable proxy to determine how diet affects microbes and the repercussions imparted onto their host. In the laboratory, the *C. elegans* diet usually consists of a single strain of *E. coli*, which then inhabits its intestine in a commensal relationship. As bacterivores, *C. elegans* have an obligatory symbiotic relationship with microbes as their food source that provide dietary nutritional supplementation, for example providing vitamins and essential amino acids^10–12^. Recent studies have shown that altering the bacterial diet can cause changes in *C. elegans* lifespan and healthspan^13–15^. Here, we have developed a *C. elegans*-*E. coli* system that allows direct modification of both the diet (*E. coli*) and the host (*C. elegans*) in response to changes in the environment. This model system takes advantage of *C. elegans* as a premiere system for studies on the aging process. Moreover, our studies highlight the effects of altering the health of *E. coli*, which is a significant component of the human intestinal microbiota.

Previously, studies on the effects of a high-glucose diet in *C. elegans* involved adding glucose either to the agar media directly or to the top of the agar medium growth plate^16–21^. This high glucose diet led to a decreased lifespan, reduced healthspan (locomotion), and changes in fat storage. In these previous studies, the additional glucose was in contact with both bacteria as well as *C. elegans*. Therefore, we questioned the mechanism of the high glucose effect: was the effect attributable to direct contact with sugar by the worm, the bacteria, or both? These previous published protocols have variations with regard to how the glucose was applied to the agar, whether the bacteria were alive or dead, how the bacteria were killed, whether the bacteria had any direct contact with the glucose^16–21^, and the age of animal exposed to the glucose^22,23^. Moreover, previous studies that tried to separate the bacterial contribution to effects on lifespan used a bacterial mutant in the major bacterial glucose transporter^20^. However, these *pts* mutants can still transport sugar at substantial, although reduced, rates^24,25^. To separate the effects of *C. elegans* consuming a glucose fed bacteria diet, here, we developed a new experimental procedure based on previous studies ^26^. In this new protocol, prior to seeding the bacteria on the plate, the bacteria are incubated with or without glucose for three days. Therefore, this protocol is unique since it allows us to control contact between the glucose and *C. elegans.*

Added dietary sugar has been associated with an alarming rise in a multitude of debilitating medical conditions including obesity, diabetes, cardiovascular and neurodegenerative diseases, such as Alzheimer’s disease, due to its impact on the generation of advanced glycation end products (AGEs). A group of heterogeneous compounds, AGEs, include proteins, lipids, and nucleic acids that become glycated as a result of exposure to sugars. Glycation results from a non-enzymatic reaction where the carbonyl group of reducing sugars is covalently coupled to proteins, lipids, and/or nucleic acids. Both exogenous and endogenous sources contribute to the levels of AGEs. Endogenous formation of AGEs occurs continuously at low levels, but exogenous sources, including consumption of a high-sugar diet, can drastically increase this pool. As humans age and in certain diseased states, AGE levels rise and are associated with increased cardiovascular risk, diabetes, chronic kidney disease, and Alzheimer's disease.

Many studies in animal model systems show that dietary consumption of exogenous AGEs contribute to oxidative stress and inflammation which could contribute to a number of chronic disease states (reviewed in^27,28^). Additionally, in cell culture dietary AGEs have been shown to affect inflammatory response^29^, but there are mixed results for the effects of dietary AGEs in human trials^27^. Emerging studies also suggest that dietary AGEs can contribute to the onset of organ damage, affect metabolic control, and therefore impact global health^28^.

We find that our *C. elegans*-*E. coli* system, where we modify the health of the microbiota through modulating *E. coli’s* dietary sugar, results in changes in lifespan and healthspan of *C. elegans*. Increased added dietary sugar for solely the bacteria results in decreased lifespan, decreased healthspan including decreased movement in liquid, and decreased oxidative stress resistance. Addition of the anti-glycation compound carnosine to the bacteria, ameliorates the negative effects of glucose in the diet on *C. elegans*. Added dietary sugar suppresses *C. elegans* oxidative stress resistance, most notably through suppression of the glutathione-S-transferase, *gst-4*, expression. Our data reveal a central role for *C. elegans gst-4* in the regulation of a high-sugar diet. Both a glucose-fed bacterial diet as well as loss of *gst-4* shorten lifespan. In addition, loss of *gst-4* blunts the animals’ response to a glucose fed bacterial diet. Taken together, our model system allows dissection of the intestinal microbiota on a level not possible in humans, with univariable analysis of both the microbiota and the host which reveals the intimate connection between oxidative stress, host responses, and a high-sugar/high AGE-producing diet.

## Results

**Figure 1:**
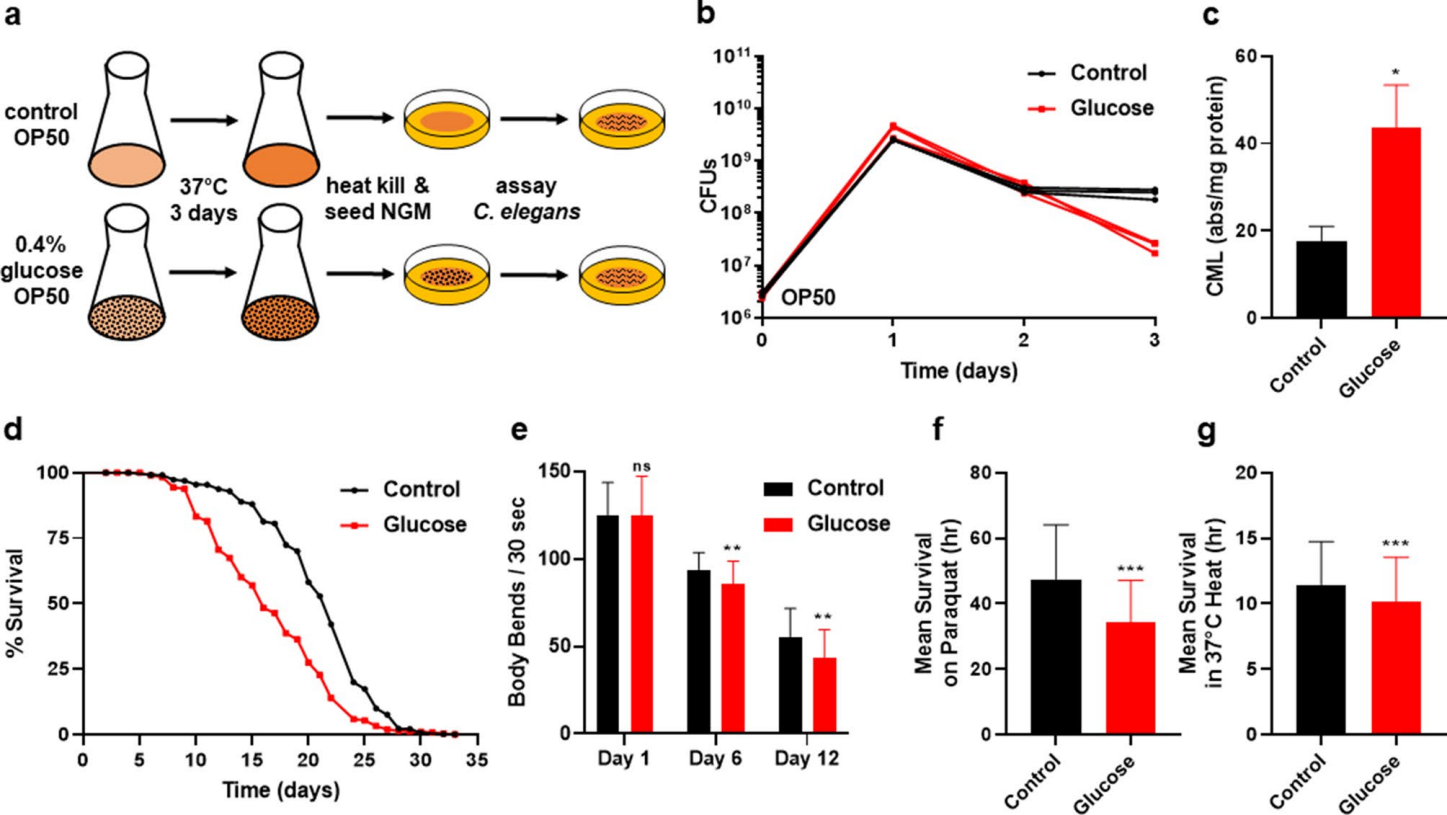
*C. elegans* consumption of glucose fed bacteria. (**a**) Experimental protocol used for OP50 *E. coli* processing of glucose and *C. elegans* assays. (**b**) Effect of 0.4% glucose supplementation on *E. coli* colony forming units over time (*P* = 0.002 at day 3). (**c**) Detection of Carboxymethyl-lysine (CML), an AGE product, by ELISA Assay performed on control and 0.4% glucose fed *E. coli*. (**d**) Lifespan of wild type *C. elegans* treated with 0% control and 0.4% glucose fed OP50 *E. coli;* Statistics in Supplementary Table S1, S2. (**e**) Healthspan—Movement in liquid/swimming/thrashing of *C. elegans* consuming control or 0.4% glucose fed *E. coli*, N = 232. (**f**) Oxidative stress resistance of wild type *C. elegans* consuming 0% control and or 0.4% glucose fed *E. coli* for 6 days, measured by mean survival on paraquat, N = 328. (**g**) Heat stress resistance (mean survival at 37 °C) of wild type *C. elegans* consuming 0% control and 0.4% glucose fed *E. coli* for 6 days, N = 366. Statistical analysis of histograms compared *C. elegans* consuming 0% control with *C. elegans* consuming 0.4% glucose fed *E. coli* at the same time point using an unpaired two-tailed t test with GraphPad Prism 8.0 (https://www.graphpad.com). Symbols as follows: (ns = not significant, *P ≤ 0.05, **P ≤ 0.005, ***P ≤ 0.001). Data shown is a compilation from at least 3 biological replicates.

Previously, studies on the effects of glucose toxicity in *C. elegans* altered several variables including the age of animal exposed to the glucose^22,23^, whether the bacterial diet was alive or dead, and the duration of and exposure time of the *E. coli* and *C. elegans* to glucose^16–21^. Additionally, the added glucose was in contact with both the *E. coli* and *C. elegans*. To determine the mechanisms leading to the effects of *C. elegans* consuming a bacterial diet high in glucose, we developed a new experimental procedure based on our previous bacterial studies^26^. In the new system, OP50 *E. coli* was inoculated in LB media supplemented with various glucose concentrations (ranging from 0 to 0.8%), and incubated for an extended period of 3 days. This was followed by heat-killing of the *E. coli* which was then seeded onto nematode growth medium (NGM) plates for physiological and biochemical assays (Fig. 1a). Heat-killing the bacterial diet prevented any possible effects of bacterial proliferation. The 3-day glucose exposure resulted in the OP50 *E. coli* exhibiting a significant decrease in colony forming units (Fig. 1b; *P* < 0.01). The optical density of the glucose-fed *E. coli* was not significantly changed, indicating that *C. elegans* were consuming similar biomass (Supplementary Figure S1a, *P* < 0.05), even though over time bacterial cells showed a significant loss of viability. We also confirmed that glucose-fed *E. coli* had elevated levels of intracellular glucose (Supplementary Figure S1b). Similar to our previous studies with a different strain of *E. coli* supplemented with glucose^26^, the 3-day incubation of OP50 *E. coli* with glucose led to an increased concentration of the AGE carboxymethyl-lysine (CML) as detected by ELISA (Fig. 1c). CML, is a major AGE and CML regulation and the extent of its accumulation is used extensively as a proxy for total AGE concentration^26,30,31^. Therefore, the OP50 *E. coli* shown in Fig. 1b supplemented with 0.4% glucose shows an increase in CML along with the loss of CFU. Together, based on our previous research^26^ and our initial studies, we selected the 3-day incubation period with supplementation of 0.4% glucose for our experimental paradigm. The experimental system presented here is unique since the glucose only directly interacts with the bacteria, never directly contacting the animals.

*C. elegans* consuming the glucose-fed *E. coli* diet show a significant reduction (~ 30%) in lifespan compared to animals fed a control diet with no added glucose (Fig. 1d, *P* < 0.01, Supplementary Table S1). Importantly, the reduced lifespan was also accompanied by a reduction in healthspan, as shown in Fig. 1e, where animals consuming the glucose fed bacterial diet, show decreased healthspan as quantified by movement in liquid/swimming/thrashing. To further evaluate health parameters, oxidative and heat stress resistance were tested on *C. elegans* after consuming the glucose-fed *E. coli* diet for 6 days (Fig. 1f–g). We selected day 6 as the time point for further analysis as this time reflected differences that could be observed prior to the onset of normal age-related effects on *C. elegans* viability. An additional phenotype of consuming a glucose-fed bacterial diet is that animals show a consistent reduction in their overall body size over time (Supplementary Figure S1c). Together, our results illustrate that consumption of the glucose-fed bacterial diet significantly reduces lifespan and healthspan which mirrors results seen in more traditional glucose supplementation experiments^16–21^. Further, these physiological effects are observed in an environment where the worms never directly encounter the additional glucose.

**Figure 2:**
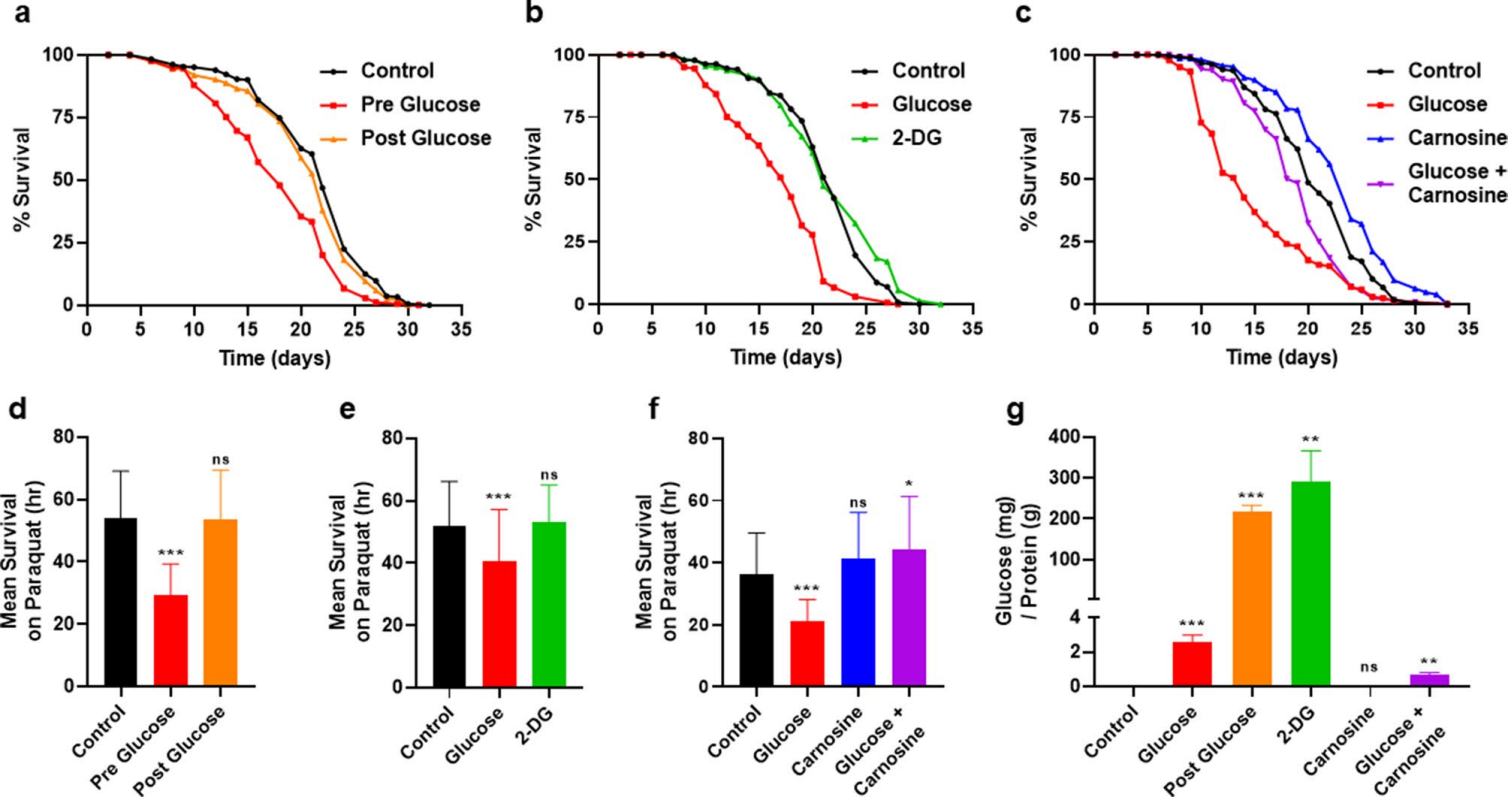
Interfering with bacterial metabolism of glucose alters its effects on *C. elegans*. (**a**) Lifespan of wild type *C. elegans* consuming either 0% control, 0.4% glucose pre culture, or 0.4% glucose post culture OP50 *E. coli*, statistics in Supplementary Table S1, S2. (**b**) Lifespan of wild type *C. elegans* consuming either 0% control, 0.4% glucose fed, or 0.4% 2-deoxy-glucose (2-DG) fed OP50 *E. coli*, statistics in Supplementary Tables S1, S2. (**c**) Lifespan of wild type *C. elegans* consuming either 0% control, 0.4% glucose, 50 mM carnosine, or 0.4% glucose + 50 mM carnosine fed OP50 *E. coli*, statistics in Supplementary Tables S1, S2. (**d**) Oxidative stress resistance of wild type *C. elegans* consuming either 0% control, 0.4% glucose pre culture or 0.4% glucose post culture OP50 *E. coli*, measured by mean survival on paraquat, N = 570. (**e**) Oxidative stress resistance of wild type *C. elegans* consuming either 0% control, 0.4% glucose, or 0.4% 2-deoxy-glucose (2-DG) fed OP50 *E. coli*, measured by mean survival on paraquat media, N = 215. (**f**) Oxidative stress resistance of wild type *C. elegans* consuming either 0% control, 0.4% glucose, 50 mM carnosine, or 0.4% glucose + 50 mM carnosine fed OP50 *E. coli*, measured by mean survival on paraquat, N = 155. (**g**) Glucose assay of the OP50 *E. coli* bacterial diet in (**a**)–(**f**), results normalized to protein concentration. Statistical analysis of histograms compared *C. elegans* consuming 0% control with *C. elegans* consuming 0.4% glucose fed *E. coli* at the same time point using an unpaired two-tailed t test with GraphPad Prism 8.0 (https://www.graphpad.com). Symbols as follows: (ns = not significant, *P ≤ 0.05, **P ≤ 0.005, ***P ≤ 0.001). Data shown is a compilation from at least 3 biological replicates.

Next, we examined how bacterial processing of glucose affects the health of *C. elegans* using three different approaches. First, we supplemented *E. coli* with glucose prior to the start of the culture (Pre glucose; method as in Fig. 1a) and compared this with glucose supplementation after the 3 day incubation (Post glucose). In the latter method (Post glucose), glucose was readily available only to *C. elegans*, in comparison to the Pre glucose diet, in which glucose was incubated only with the *E. coli.* As shown in Fig. 2a, lifespan was shortened only when *E. coli* was able to process the glucose in the Pre-glucose treatment. Remarkably, although the post glucose supplementation had a higher amount of glucose available to the animal (80×; Fig. 2g), consumption of the Post glucose diet showed no change in lifespan (Fig. 2a). In addition to the effect on lifespan, oxidative stress resistance was reduced only in the Pre glucose diet (Fig. 2d). We also assayed healthspan as measured by movement of the animals in liquid as animals age. Movement of the animals in liquid required a greater time to observe any effect and was not substantially reduced by the Post glucose supplementation until day 12 (Supplementary Figure S2a). Therefore, it is the processing of glucose by the bacteria that decreases lifespan, oxidative stress resistance, and locomotion-healthspan.

A second method to examine the effects of bacterial metabolism of glucose on *C. elegans* was the use of the synthetic glucose analog 2-deoxy-D-glucose (2-DG). When consumed, 2-DG is phosphorylated by hexokinase which then cannot be further processed and therefore is used as a glycolytic inhibitor. We treated the OP50 *E. coli* with 2-DG for 3 days, similar to previous experiments. *C. elegans* consuming OP50 *E. coli* supplemented with 2-DG exhibit a normal lifespan without changes in either resistance to oxidative stress or healthspan (movement in liquid; Fig. 2b,e, and Supplementary Figure S2b). Thus, consuming the 2-DG fed bacteria diet results in phenotypes opposite to those seen with the glucose fed bacterial diet.

Third, to further elucidate the connection between bacterial metabolism of glucose and host health, we supplemented the bacterial diet with the dipeptide carnosine in combination with glucose. We have previously observed that the anti-glycation properties of carnosine reduces the toxic effects of glucose in *E. coli*^26^. As shown in Fig. 2c, glucose plus carnosine-fed *E. coli* significantly extends *C. elegans* lifespan and ameliorates the effect of added glucose alone (*P* < 0.01). Interestingly, there is little to no effect of carnosine on *C. elegans* when the compound is mixed within the agar of the plate then seeded with heat-killed *E. coli* (Supplementary Figure S2c). Additionally, glucose concentrations within the *E. coli* are decreased by 3× when the glucose-fed *E. coli* are supplemented with carnosine (Fig. 2g). *C. elegans* consuming this glucose and carnosine diet are protected from the decrease in oxidative stress resistance that occurs in glucose supplementation alone (Fig. 2f). Therefore, across three different methods, we reveal that the bacterial processing of glucose dictates *C. elegans* lifespan and healthspan.

**Figure 3:**
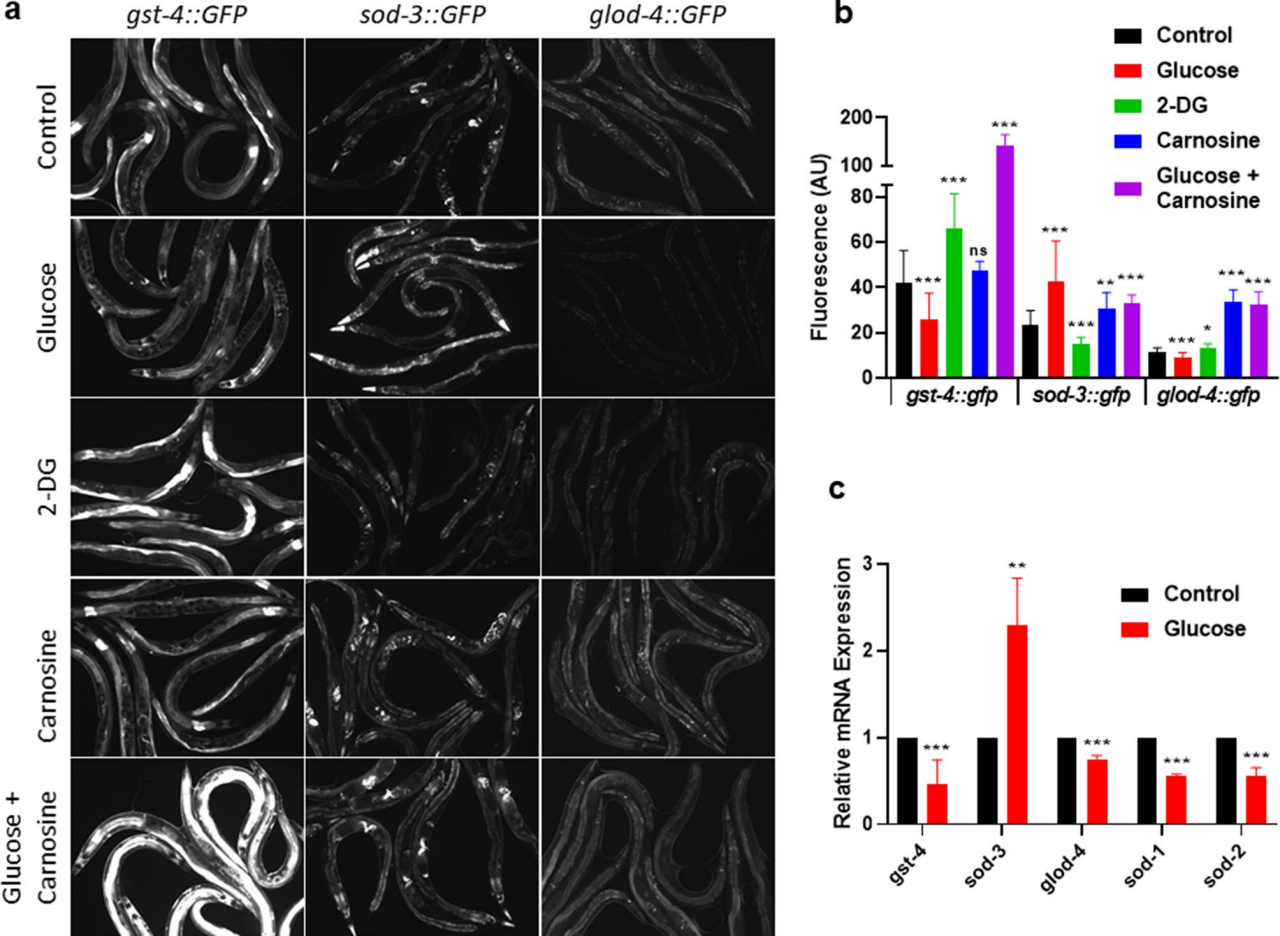
Effects of bacterial metabolism of glucose on host *C. elegans* gene expression. (**a**) Fluorescent imaging of transgenic *C. elegans* *gst-4::gfp*, *sod-3::gfp*, or *glod-4::gfp*, after 6 days consuming either 0% control, 0.4% glucose, 0.4% 2-deoxy-glucose (2-DG), 50 mM carnosine, or 0.4% glucose + 50 mM carnosine fed *E. coli* for 6 days. See methods for complete genotype of transgenic *C. elegans*. (**b**) Fluorescence quantification of animals in (**a**): transgenic *C. elegans* *gst-4::gfp*, *sod-3::gfp*, or *glod-4::gfp*, consuming either 0% control, 0.4% glucose, 0.4% 2-deoxy-glucose (2-DG), 50 mM carnosine, or 0.4% glucose + 50 mM carnosine, fed *E. coli* for 6 days, N = 336. (**c**) RTqPCR of wild type *C. elegans* consuming either 0% control or 0.4% glucose fed *E. coli* for 6 days. Statistical analysis of histograms compared *C. elegans* consuming 0% control with *C. elegans* consuming 0.4% glucose fed *E. coli* at the same time point using an unpaired two-tailed t test with GraphPad Prism 8.0 (https://www.graphpad.com). Symbols as follows: (ns = not significant, *P ≤ 0.05, **P ≤ 0.005, ***P ≤ 0.001). Data shown is a compilation from at least 3 biological replicates.

Animals consuming the glucose-fed bacteria diet exhibit a shortened lifespan and decreased stress resistance. Therefore, we next examined whether the expression levels of key genes involving environmental defenses could account for these observed phenotypes using both transgenic GFP animals as well as RT-qPCR. We used known stress-related transgenic GFP strains to observe any potential changes in the dynamics of expression associated with consumption of glucose-fed bacteria within the host over time. To determine the downstream signaling pathway in the host that responds to the glucose-fed bacteria diet, we screened distinct pathways induced by oxidative stress *(gst-4, sod-3)*^32^, heat stress *(hsp-16.2)*, the mitochondrial unfolded protein response (UPR) *(hsp-6)*^33,34^, the glyoxalase system *(glod-4)*^35^, the transcription factor *skn-1*^36^, and the insulin/IGF-1 signaling pathway transcription factor *C. elegans* FOXO/*daf-16*^37–40^ (Fig. 3a and Supplementary Figures S3a–b, S4a–d, S5a–b).

Notably, as shown in Fig. 3a, we observed blunted expression of both *gst-4::GFP* and *glod-4::GFP* when animals consumed the glucose-fed bacteria, as well as an increase in *sod-3::GFP*. To remove any bias associated with the visual assay, we quantified the fluorescence of those animals (Fig. 3b). Then, to confirm the validity of our findings, we performed RT-qPCR on wild-type animals under the same conditions. We find consistent gene expression changes when assayed by RT-qPCR (Fig. 3c).

Next, we tested whether gene expression changes paralleled our findings in Fig. 2 with the use of the glucose analog 2-DG, which did not alter *C. elegans* lifespan or healthspan. As shown in Fig. 3a, we observed gene expression levels opposite to glucose when animals were fed a bacterial diet supplemented with 2-DG. This finding was confirmed with fluorescence quantification (Fig. 3b). Therefore, in response to a glucose-fed diet, we observed a decrease in lifespan and healthspan associated with a downregulation of several key detoxification genes.

Further, we examined GFP expression changes in *C. elegans* when fed a diet consisting of *E. coli* fed carnosine alone or in combination with glucose, as carnosine ameliorated the shorter lifespan induced by glucose (Fig. 2). *C. elegans* consuming carnosine-fed *E. coli* exhibit a mild increase in lifespan and also show a slightly higher level of *gst-4::GFP*, *sod-3::GFP*, and *glod-4::GFP* expression (Fig. 3a,b). When both carnosine and glucose are fed to the bacteria, lifespan is greater than glucose alone and *gst-4::GFP* fluoresces significantly brighter, while *sod-3::GFP* and *glod-4::GFP* show similar expression to carnosine alone (Fig. 3a,b; *P* < 0.01). Furthermore, *C. elegans* consuming carnosine-fed *E. coli* exhibit longer lifespan, protection from the decline in oxidative stress resistance and changes in gene expression when compared to consuming the glucose-fed bacterial diet insulin/IGF-1signaling (IIS) (Figs. 2f, 3a,b).

We also screened two signaling pathways: Detoxification- *skn-1* (*C. elegans* NRF2) and IIS- *daf-16* (*C. elegans* FOXO) for gene expression changes in response to consumption of the glucose fed bacterial diet using both transgenic GFP animals as well as RT-qPCR. As shown in Supplementary Figure S3a, the glucose-fed bacterial diet did not change either expression levels of *skn-1::GFP* or nuclear translocation. The *skn-1::GFP* results were consistent with the RTqPCR results shown in Supplementary Figure S3b where mRNA of *skn-1* was not significantly changed with the glucose-fed bacterial diet. Since *skn-1* showed no changes in expression in response to the glucose-fed bacterial diet, we further tested one of its target genes, *gcs-1*, often used as a surrogate for *skn-1*^41^. There was neither a change in *gcs-1::GFP* expression nor *gcs-1* mRNA levels (Supplementary Figures S3a and S3b). Additionally, the heat stress marker *hsp-16.2* was consistently downregulated with the glucose-fed bacterial diet as determined by both GFP fluorescence and mRNA expression (Supplementary Figures S3a and S3b).

We also examined the IIS pathway in response to the glucose-fed bacterial diet initially with nuclear translocation of *daf-16a::GFP.* We did not observe any changes in nuclear translocation for *daf-16a::GF*P but did observe a significant reduction in total fluorescence per animal (Supplementary Figures S4a and S4b; *P* < 0.01) and RT-qPCR showed a 20% increase in *daf-16* mRNA (Supplementary Figure S4c). Further analysis of DAF-16 activity was performed by quantifying expression of 14 well-known transcriptional targets of DAF-16^37,38,42^. Of these DAF-16 targets, 10/14 were significantly changed with the glucose fed bacteria; *sod-3*, *cpr-2*, *ctl-1*, and *dod-6* upregulated; *hsp-12.6*, *ZK742.4*, *fat-7*, *scl-1*, *ctl-2*, and *dod-3* downregulated (Supplementary Figure S4d, *P* < 0.01). Therefore overall, our data suggest that DAF-16 is partly involved in the response to a glucose-fed bacterial diet.

We observed a consistent upregulation of *sod-3* and *sod-5* with the glucose-fed bacterial diet, which presumably should increase resistance to oxidative damage^43^. However, a recent report by Dues et al^44^ examined the total superoxide dismutase capacity of *C. elegans*. They found that *sod-3* and *sod-5* together only account for 1.7% of all *sod* expression while *sod-1*, *sod-2*, and *sod-4* together amass 98.2% of the total *sod* mRNA. In accordance with these findings, we mathematically extrapolated our RTqPCR results onto the published expression ratios to estimate total *sod* expression (Supplementary Figures S5a–b). Animals consuming glucose fed bacteria show a ~ 50% decrease in *sod-1* and *sod-2* expression which overshadows any increase in *sod-3* expression. Therefore overall, animals consuming the glucose-fed bacterial diet exhibit a reduction of *sod* expression to only 60% of total wild type *sod* mRNA capacity (Supplementary Figure S5b). Therefore, across multiple experimental paradigms, our data confirm that bacterial processing of glucose promotes a decrease in lifespan as well as a reduction in health, and stress resistance. These phenotypical changes are accompanied by differential regulation of glyoxalase, glutathione-S-transferases, and superoxide dismutase—all of which should enzymatically detoxify the glycolytic effects of glucose.

Our data reveal that glucose-fed bacteria which are high in CML (AGEs) promote an environment that causes animals to exhibit signs of oxidative stress including strong and consistent suppression of *gst-4* (glutathione transferase-4) involved in the regulation of the Phase II oxidative stress response^45^. Analysis of transcriptional activation of *gst-4* is often used as a proxy for oxidative stress tolerance^46^. Therefore, we next examined a loss of function allele of *gst-4* generated by CRISPR/Cas9.

**Figure 4:**
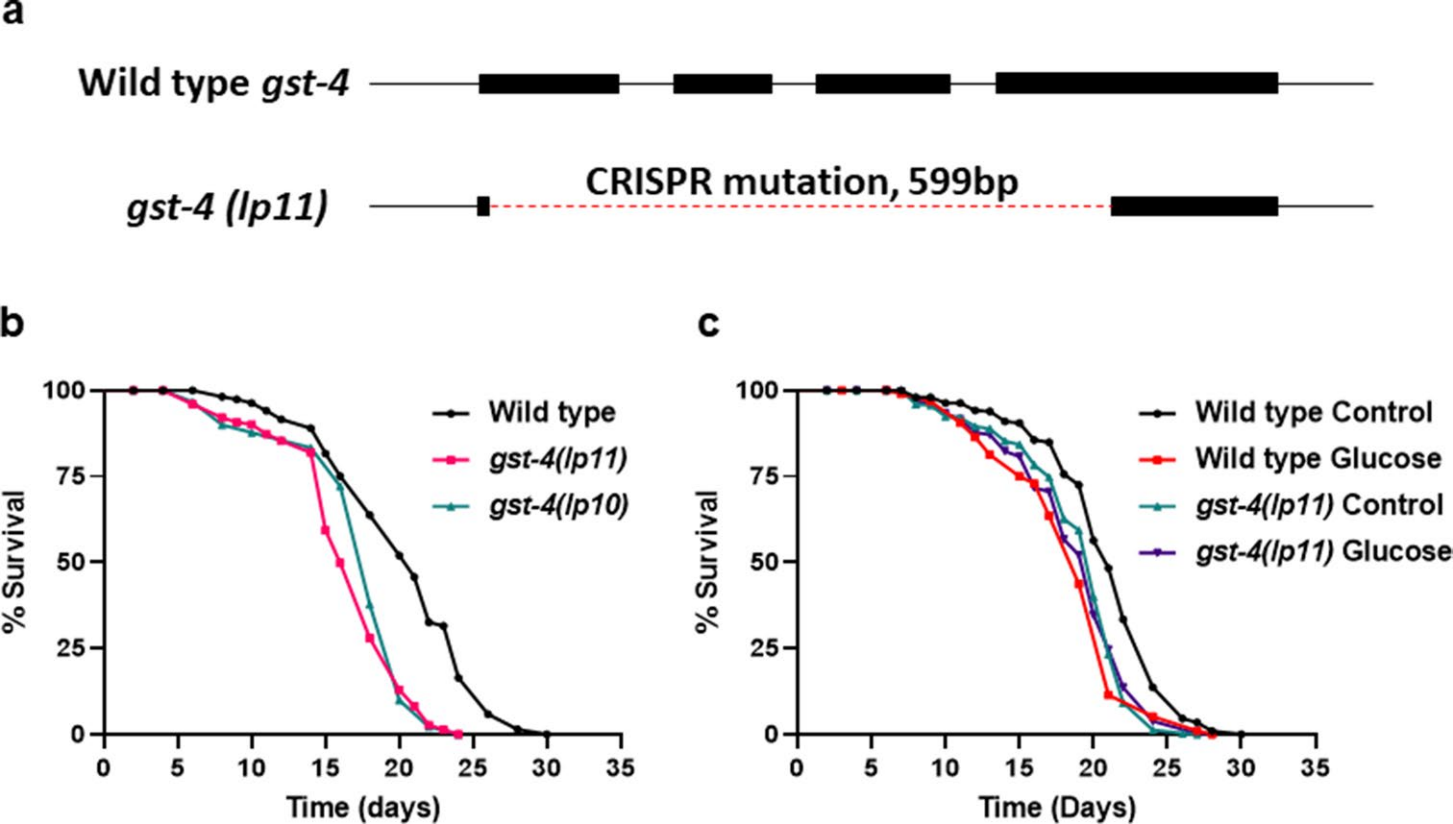
Generation and characterization of *C. elegans gst-4* mutant. (**a**) Diagram of DNA sequences of wild type *C. elegans* genomic *gst-4* and the *gst-4(lp11)* mutant. Lines represent introns, rectangles represent exons, and dashed line represents the *lp11* allele CRISPR deletion. (**b**) Lifespan of wild type, *gst-4(lp10)*, and *gst-4(lp11)*, statistics in Supplementary Table S1, S2. (**c**) Lifespan of wild type and *gst-4(lp11)* mutants consuming either 0% control or 0.4% glucose fed OP50 *E. coli*, statistics in Supplementary Tables S1, S2.

We generated two independent isolates of the *gst-4* mutation, *lp10* and *lp11* by CRISPR/Cas9. As shown in Fig. 4a, sequencing *lp11* revealed a deletion of 599 bp and spans all 4 exons of wild type *gst-4*. Next, we tested the *gst-4* mutants for lifespan and show that the *gst-4* mutation results in a decrease in lifespan (Fig. 4b). We further examined whether loss of *gst-4* interfered with the effects of the glucose fed bacterial diet. As shown in Fig. 4c, mutation in *gst-4* abrogates the response to the glucose fed bacterial diet. Therefore, our results indicate that *gst-4* is necessary to respond to a glucose-fed bacterial diet. Together, our data reveal that dietary AGEs promote oxidative stress in the host dependent on the Phase II oxidative stress response.

## Discussion

As bacterivores, the diet of *C. elegans* in the laboratory typically consists of a single *E. coli* strain, which then inhabits its gut in a commensal relationship. Here, we test the *C. elegans-E. coli* interaction in response to changes in the dietary environment, specifically with the addition of glucose. Previous studies examining a high glucose diet involved several different methods. A given percentage of glucose, typically 2%, is either added to the NGM agar before cooling or added to the top of the NGM agar filled plate after it has cooled. Bacteria, the diet for *C. elegans,* would then be spread onto the NGM agar plate. In such an experimental paradigm, there is a three-way interaction: *E. coli* is exposed to the glucose, *C. elegans* is exposed to the glucose, and *C. elegans* consumes the *E. coli* exposed to the glucose^16–21,42,47,48^. As shown in our model in Supplementary Figure S6, with these methods, glucose has both direct and indirect contact with *C. elegans*.

In contrast, our *C. elegans-E. coli* system specifically examines bacterial processing of glucose and its effect on *C. elegans*. Addition of glucose to the bacterial culture precedes any exposure to *C. elegans*. These glucose-fed bacteria are then seeded onto the NGM agar plate thereby illustrating the indirect effect of glucose on *C. elegans.* With this method*,* we find that *C. elegans* consuming the glucose-fed *E. coli* diet have a 24% reduction in lifespan and a reduction in healthspan (locomotion) and oxidative stress resistance (Fig. 1, Supplementary Table S1). Therefore, both the indirect and direct effects of glucose result in poor health as animals age^16–21^.

Our data also illustrate that the duration of direct contact between the glucose and the bacteria is critical. As shown in Figure S7, similar to findings by Lee et al^20^, pre-incubating the bacteria overnight (16 h) does not confer any changes in lifespan (Supplementary Figure S7). Based on our previous studies^26^, we considered that perhaps the *E. coli* pre-exposure to glucose required a longer incubation. Subsequently, we chose a 3-day incubation with glucose prior to seeding the *E. coli* onto the NGM plate. In effect, our *C. elegans-E. coli* system reduces the variability of bacterial metabolism by imposing a consistent window in which bacteria process glucose. Addition of glucose to the bacterial culture allows processing by the bacteria to occur preceding any exposure to *C. elegans*.

Our data reveal that the specific bacterial processing of glucose negatively affected the health of *C. elegans* within three different methods to separate, inhibit, and suppress bacterial processing (Fig. 2). First, we supplemented the *E. coli* culture with the same 0.4% concentration of glucose after the bacterial diet was heat-inactivated. This direct application of glucose had no effect on *C. elegans* lifespan perhaps since this is a relatively low amount of glucose when compared to previous reports utilizing 2% per plate. However, when processed by the bacteria, 0.4% glucose drastically changes the health of the host. Secondly, we incubated the *E. coli* with glucose analog 2-DG to inhibit glycolysis within the bacteria. In contrast to glucose fed bacteria, 2-DG fed bacteria result in healthier animals with a normal lifespan. In a third set of experiments, we incubated the *E. coli* culture with carnosine, an anti-glycation compound, that has the ability to lower glucose toxicity through reducing the accumulation of AGEs^26^. We observed that carnosine alone, as well as carnosine in combination with glucose, resulted in healthier animals compared with consumption of a glucose-fed bacterial diet. Therefore, across three methods, our data reveal that the bacterial processing of glucose dictates the phenotypes of the animals consuming this diet.

Consistently, the high AGE *E. coli* diet significantly shortened mean lifespan of *C. elegans*. However, when fed the glucose and carnosine treated *E. coli*, lifespan was reduced only by ~ 7% (Supplementary Table S1). Additionally, when applied directly to the NGM media with heat-killed bacteria, carnosine had little to no effect on *C. elegans* lifespan. Thus, benefits were only observed when carnosine was processed by the bacteria. Carnosine treated *E. coli* also increased *C. elegans* healthspan as measured by resistance to oxidative stress, both alone and in the presence of glucose. This additional oxidative stress resistance may be attributed to the ability of carnosine to reduce the glucose burden of the bacteria by lowering total AGEs consumed by *C. elegans*. Together, these results suggest that carnosine used as a prebiotic intervention may have the potential to effect resident microbiota and alleviate dietary glucose related oxidative stress.

Previous studies in mammals have linked oxidative stress to consumption of a high AGE diet^28,49–51^. We show that when animals consume the glucose-fed bacterial diet, they are subject to high levels of oxidative stress. We surveyed a broad panel of stress reporter genes when animals are subject to the glucose-fed bacterial diet (Fig. 3, Supplementary Figures 3–5). Animals consuming the glucose-fed bacterial diet exhibit a down regulation of *gst-4*, both in *gst-4::GFP* expression and mRNA levels. Interestingly, our data show that the microbial processing of glucose inhibits the expression of *gst-4,* as 2-DG treated bacteria did not confer this same effect. Further, our analyses reveal that the glucose-fed bacterial diet promotes an environment of constant oxidative stress such that when *C. elegans* age on this diet, they become more susceptible to oxidative stress. Furthermore, our data reveal that loss of *gst-4* prevents the animal from responding to the glucose-fed bacterial diet (Fig. 4). It should be noted that we examined multiple other glutathione-S-transferases including *gst-10, gst-29* and found that they were all downregulated. This indicates the possibility that the bacterial processing of glucose suppresses the animal’s ability to respond to this oxidant rich environment, which may perpetually cause chronic vulnerability to oxidants.

**Figure 5:**
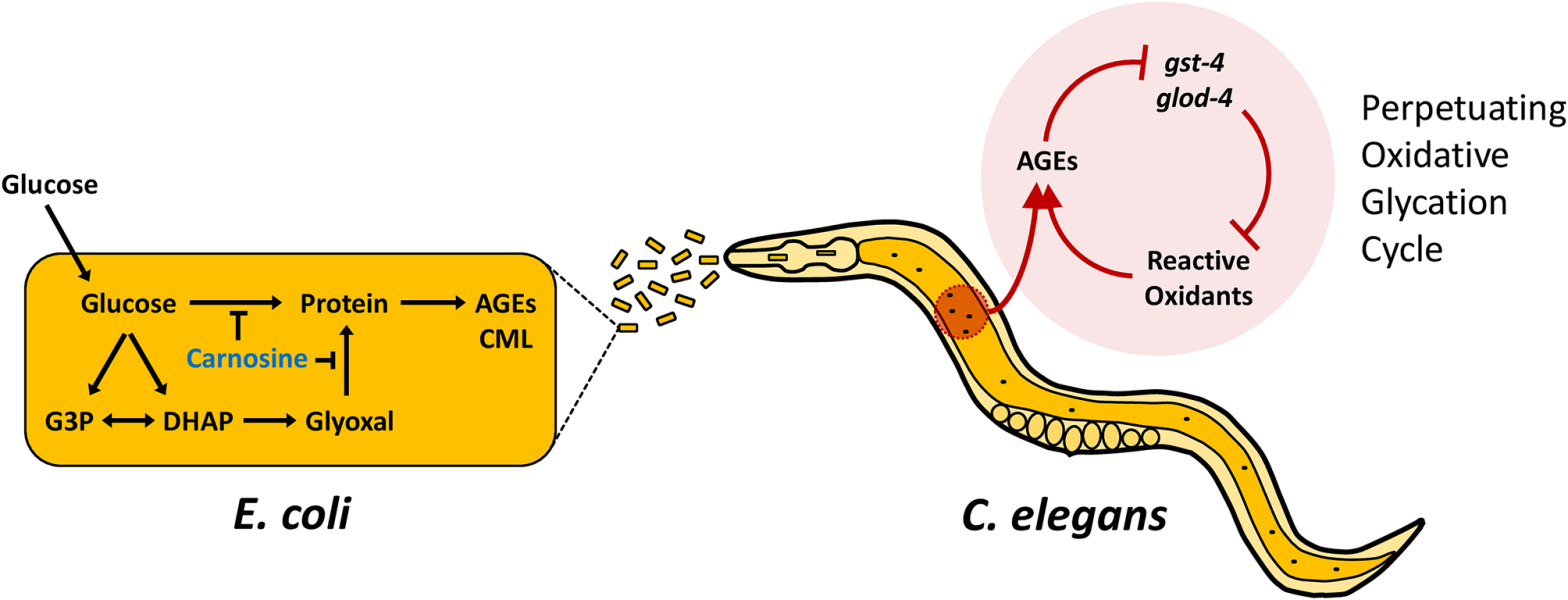
Model of *E. coli* glucose processing effects on *C. elegans.* Glucose is processed/metabolized within the *E. coli* to produce AGEs. Carnosine supplementation abrogates this formation. Consumption of the glucose-fed bacteria with successive digestion and absorption leads to *C. elegans* transcriptional changes. High AGEs from the diet suppress *gst-4* and *glod-4*; two genes which function to prevent reactive oxidants such as glyoxal and methylglyoxal from nonenzymatically binding with proteins and forming more AGEs. Thus, the inhibition of *gst-4* and *glod-4* cause a perpetuating oxidative glycation cycle, further limiting *C. elegans* ability to respond to AGEs/oxidants.

Our data suggest a model shown in Fig. 5. Uptake of glucose by the bacteria leads to increased processing of glucose and results in more bacterial-generated AGEs. Consumption of this glucose-fed bacterial diet high in AGEs promotes a transcriptional response including suppression of *gst-4* and *glod-4*. Enzymes such as *gst-4* and *glod-4* function to prevent reactive oxidants such as glyoxal and methylglyoxal from nonenzymatically binding to proteins forming more AGEs^32,35^. Inhibition of *gst-4* and *glod-4* result in a perpetuating oxidative glycation cycle that results in even more AGEs produced and further increase of oxidative stress.

We suggest that using our *C. elegans*-*E. coli* system, it is possible that we mimic dietary effects on bacteria within the human digestive tract. We use a 3-day bacterial culture supplemented with glucose to analyze the effect onto its host. We suggest this is similar to the entrenched bacteria within the human digestive tract that are dependent on nutrient intake by their host. Interestingly, the physiological manifestation of Crohn’s disease is characterized by bacterially derived persistent inflammation of the intestine and colon. In fact, recent clinical studies find that patients suffering from Crohn’s disease and Irritable Bowel Syndrome exhibit oxidative stress^52^. Therefore, the blunted transcriptional response observed when animals consume the prebiotic carnosine with the glucose-fed bacteria could aid future models of the dynamics involved in persistent bacterial inflammation in cases of Crohn’s disease and Irritable Bowel Syndrome.

Taken together, our data show that bacterial processing of glucose resulted in reduced lifespan and reduced stress resistance in their host even though the host never directly encountered the additional glucose. High levels of AGEs in the *C. elegans* diet are accompanied with downregulation of stress response genes. This overall transcriptional response by *C. elegans* in response to consumption of glucose-fed bacteria is protected by the preventative mechanisms of action of carnosine. Consumption of the high glucose/highly glycated/increased AGEs diet shortens lifespan, reduces healthspan and promotes oxidative stress.

## Methods

### Strains

All *C. elegans* strains were maintained at 16 °C using standard *C. elegans* techniques except where indicated^53^. Strains used in this study: N2, LGIV—HT2335 *gst-4(lp10)*, HT2336 *gst-4(lp11)*.

The following transgenes were used: TJ356 [*daf-16a::gfp*], LD1008[ldEx9 [*skn-1::GFP* + *rol-6(su1006)*]], BC15643 [*dpy-5(e907)* I; sEx15643[*rCes C16C10.10::GFP* + *pCeh361*]], CL2166 [dvIs19 [*(pAF15)gst-4*_*p*_*::GFP::NLS*] III.], CF1553 [muIs84 [P*sod-3::GFP*]], CL2070 [dvIs70[P*hsp-16.2::GFP* + *rol-6(su1006)*]], LD1171 [ldIs3 [P*gcs-1::GFP* + *rol-6(su1006)*]], Some strains were provided by the CGC, which is funded by NIH Office of Research Infrastructure Programs (P40 OD01044).

### Lifespan assay

All lifespan assays were performed at 20 °C. Strains were semi-synchronized by allowing gravid adults to lay eggs on standard NGM plates overnight, and animals developed for several days until they reached L4. Then, ∼100 L4s were transferred to NGM or treatment plates and kept at 20°(∼33 animals per plate). Animals were scored by gently tapping with a platinum wire every 2–3 days. Animals that did not respond were scored as dead. Animals that crawled off the plate, bagged, or died from vulva bursting were censored from the analysis. Each figure shows data collected from all trials. The mean lifespan, standard deviation, and P values were calculated via two-tailed unpaired t tests (Supplementary Table S1). Survival graphs and statistical analyses were produced using GraphPad Prism 8.0 (https://www.graphpad.com).

### Glucose/carnosine fed *E. coli* diet

Standard Nematode Growth Media (NGM)^53^ was autoclaved at 121 °C for 25 min, then cooled at room temperature (RT) and 100 µg/mL ampicillin (Fisher Scientific) was added prior to pouring plates. Plates dried for 3 days at RT before seeding with *E. coli*. The OP50 *E. coli* was grown for 3 days in LB only (control), or LB supplemented either pre-culture or post-culture with 0.4% D-(+)-glucose (Sigma), 0.4% 2-Deoxy-D-glucose (Sigma), and/or 50 mM L-carnosine (Sigma). After 3 days, the *E. coli* cultures were subjected to a 65 °C heat bath for 30 min to inhibit further growth and then seeded onto the NGM plate. The *E. coli* OP50 diet was streaked onto an LB agar plate and cultured at 37 °C overnight to confirm lack of growth. Plates were stored at 4 °C until use.

### *E. coli* fitness (survival) assay

Overnight cultures were inoculated from frozen stocks. 100 μl of each strain was spread on each plate in triplicate and allowed to dry by evaporation. At each time point, the number of viable cells was determined by “coring” a section of the lawn with a sterile Pasteur pipet (inner diameter ~ 1.5 mm), resuspending each core in 50 μl of LB, and titering by serial dilution and plating onto LB medium; the limit of detection is < 100 cfu/ml^54,55^.

### *E. coli* AGE assay

ELISA was used to detect carboxymethyl-lysine levels as in Pepper et al^26^ with 100 μl of supersensitive, 3,3′,5,5′-tetramethylbenzidine (TMB) (Sigma) used as the color reagent and read at 630 nm on a BioTek ELx808 absorbance reader.

### Glucose assay

OP50 *E. coli* was grown for 3 days in LB only (control), or LB supplemented either pre-culture or post-culture with 0.4% D-(+)-glucose (Sigma), 0.4% 2-Deoxy-D-glucose (Sigma), and 50 mM L-Carnosine (Sigma). After 3 days, the *E. coli* cultures were subjected to a 65 °C heat bath for 30 min to inhibit further growth. Samples were diluted in reaction buffer to accurately detect glucose levels and frozen at − 20 °C until use. Glucose amount was determined using the Amplex Red Glucose/Glucose Oxidase Assay Kit (Invitrogen), in triplicate. Protein concentration was then measured with Pierce Coomassie Plus (Bradford) Assay Kit (Thermo Scientific). Glucose levels were standardized to protein levels, and then used to graph over three replicate experiments using GraphPad Prism 8.0 (https://www.graphpad.com).

### RNA extraction and RT-qPCR

Approximately 200 synchronized L4 stage animals were transferred to treatment plates and grown at 20 °C for 6 days, then washed off the plates with M9 buffer and rinsed twice with DEPC-treated water. Total RNA was isolated using TRIzol Reagent with the Direct-zol RNA MiniPrep (Zymo Research). After RNA extraction, first-strand cDNA was synthesized from 1.0 μg of total RNA using dNTPs, Oligo(dT)_12–18_ and SuperScript II Reverse Transcriptase (Invitrogen). Quantitative PCR was done with an Applied Biosystems StepOne Plus Real-Time PCR system with Power SYBR Green PCR Master Mix (Applied Biosystems) per the manufacturer’s instructions, with triplicates done for each of three biological replicates, and *act-1* used as the endogenous control for relative expression normalization. Sequences of primers can be found in Supplementary Table S3. Specificity of PCR amplification was determined by the melting curve for each reaction. The threshold cycle (CT) for each primer set was automatically determined by StepOne Software v2.3 (https://www.thermofisher.com/us/en/home/technical-resources/software-downloads/StepOne-and-StepOnePlus-Real-Time-PCR-System.html). Relative fold changes of gene expression were calculated using the 2^−△△^Ct method. The RQ values were then used to graph relative gene expression over at least three replicate experiments using GraphPad Prism 8.0 (https://www.graphpad.com).

### Healthspan: body bends/movement in liquid media/thrashing/swimming

Synchronized L4 stage animals were transferred to treatment plates and incubated at 20 °C for 1, 3, 6, or 12 days. On the experimental day, individual animals were picked onto an unseeded NGM plates, then 30 μL of M9 buffer was pipetted onto each animal. After 5 s, the number of body bends was recorded over 30 s using a mounted DMK 21AF04 camera (The Imaging Source) outfitted onto a dissecting microscope. At least 20 animals were recorded per strain or treatment and the average body bends per minute for each of the samples was calculated and graphed using GraphPad Prism 8.0 (https://www.graphpad.com).

### Resistance to oxidative stress

Paraquat plates were made by adding 1 mL of 250 mM paraquat solution to corresponding treatment plates. Then plates were put on a shaker for 1 h, followed by 1.5 h in a laminar flow hood to ensure plates were dry with the paraquat evenly distributed. Synchronized L4 stage animals were transferred to treatment/paraquat plates and incubated at 20 °C for 6 days. Animals were then transferred to paraquat plates at 20 °C and scored twice daily for survival. Animals were touched with a platinum wire, and those that did not respond were scored as dead. Two independent replicates were performed, and the mean survival calculated using GraphPad Prism 8.0 (https://www.graphpad.com).

### Resistance to heat stress

Synchronized L4 stage animals were transferred to treatment plates and incubated at 20 °C for 6 days. Animals were transferred to 37 °C and then scored every 2 h for survival. Animals were touched with a platinum wire, and those that did not respond were scored as dead. Two independent replicates were performed, and the mean survival was calculated using GraphPad Prism 8.0 (https://www.graphpad.com).

### Fluorescence imaging and quantification

Synchronized L4 stage animals were transferred to treatment plates and incubated at 20 °C for 1, 3, 6, or 12 days. On the experimental day, 10–12 animals were then picked onto 2% agarose pad on a glass slide and immobilized with 100 mM NaN_3_. Fluorescence imaging of GFP was done using a Zeiss Axioskop 2 plus fitted with a Hamamatsu ORCA-ER camera and a FITC filter. Images taken were in grayscale. Quantification of GFP expression was performed using ImageJ Software 1.52a (https://imagej.nih.gov/ij/index.html). In the photos, each worm at least 95% was in frame was outlined by hand and then measured for minimum, maximum, and average pixel intensity within the defined area. The minimum pixel intensity recorded was then subtracted to remove background fluorescence interference. The average pixel intensity per worm compiled across 2 or more batch experiments was then plotted using GraphPad Prism 8.0 (https://www.graphpad.com).

### CRISPR/Cas9 generation of a *C. elegans* mutant

Guide RNA sequences were designed using http://crispor.tefor.net^56^ and genome editing was performed as described previously^57,58^. Cas9, tracrRNA and two crRNAs targeting the coding region of *gst-4* were injected as ribonucleoprotein (RNP) complexes into gonads of N2 hermaphrodites. Using Prf4:*rol-6(su1006)* as injection marker, F1 Roller progeny were cloned and genotyped for deletions. Genotyping was performed with oligos flanking the guide binding sites to identify mutants that lack the entire region between the crRNA target sites. Genotyping PCR primer sequences were designed utilizing the aid of IDT DNA PrimerQuest tool and were manufactured by Invitrogen. Two such deletion mutants were isolated as heterozygotes and later homozygosed to obtain *gst-4(lp10)* and *gst-4(lp11)*. After PCR, the sequence was confirmed with DNA sequencing performed by GENEWIZ, Inc.

## Supplementary Information

Supplementary Information
