# Supplementary material for: Bacterial processing of glucose modulates C. elegans lifespan and healthspan
Source: Sci Rep. 2021 Mar 15;11:5931. doi: 10.1038/s41598-021-85046-3 (PMC7971010; doi:10.1038/s41598-021-85046-3)
Supplement: Supplementary file 1 — Supplementary Information [file 41598_2021_85046_MOESM1_ESM.pdf]

Bacterial processing of glucose modulates *C. elegans* lifespan and healthspan

Supplementary Figures and Tables

Samuel F. Kingsley[1α], Yonghak Seo[1α], Calista Allen[2], Krishna S. Ghanta[3], Steven Finkel[2], and Heidi A. Tissenbaum[1,4*]

[1]Department of Molecular, Cell and Cancer Biology, University of Massachusetts Medical School, Worcester, MA 01605

[2]Molecular and Computational Biology Section, Department of Biological Sciences, University of Southern California, Los Angeles, CA, 90089, USA

[3]RNA Therapeutics Institute, University of Massachusetts Medical School, Worcester, MA, 01605, USA

[4]Program in Molecular Medicine, University of Massachusetts Medical School, Worcester, MA, 01605, USA

[α]Co-first author

*Corresponding author: Heidi.Tissenbaum@umassmed.edu

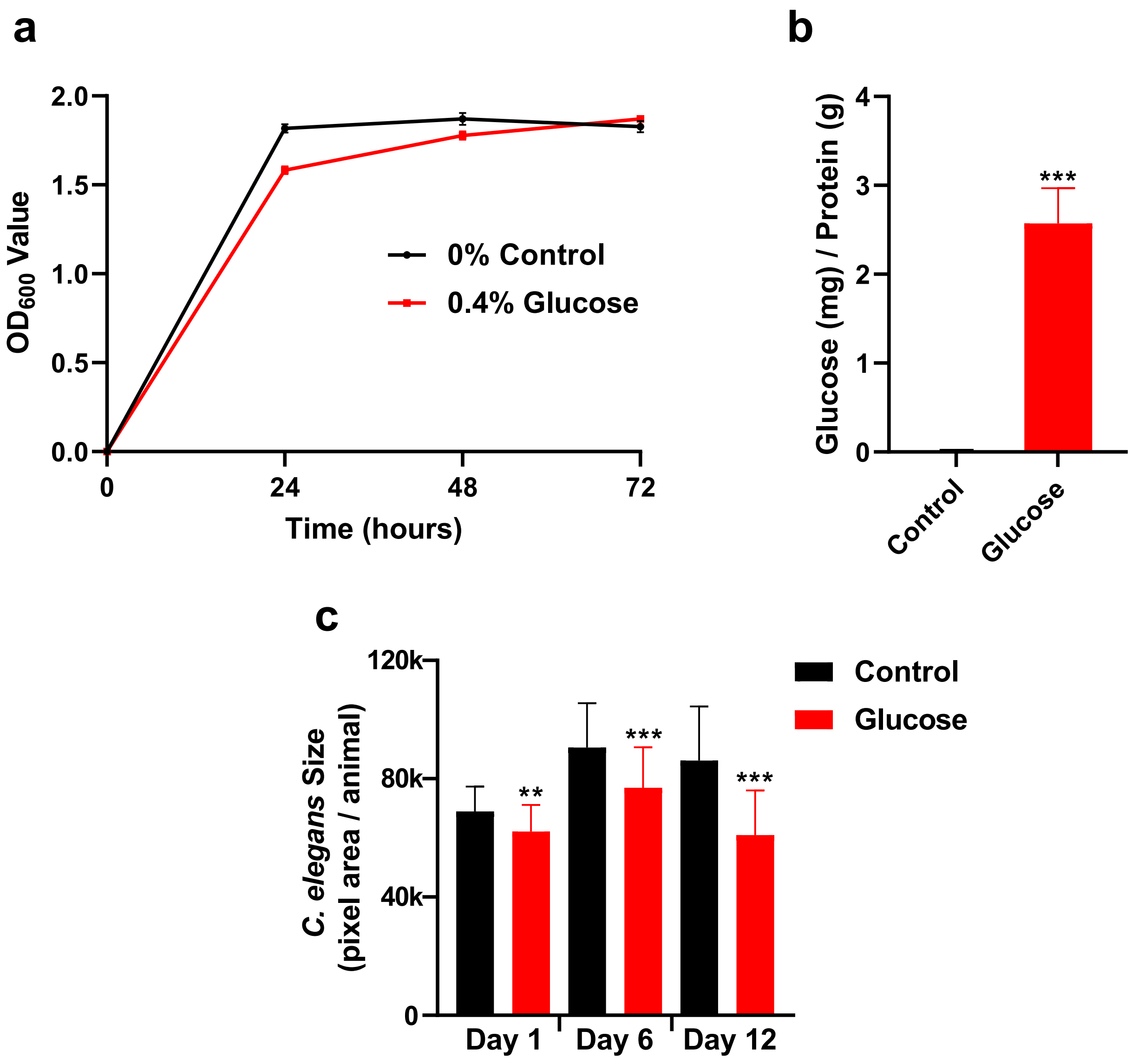

Supplementary Figure S1. Effects of Adding Glucose Directly to *E. coli*. (a, b–*E. coli,* c–*C. elegans)* (a) Optical Density (600nm) of 0% control and 0.4% glucose fed OP50 *E. coli* over 24 hr (P ≤ 0.001 ***), 48 hr (P ≤ 0.05*) and 72 hr (P > 0.05^{ns}). (b) Glucose assay of 0% control and 0.4% glucose fed OP50 *E. coli* after 3 days in LB, normalized to protein concentration. (c) *C. elegans* body size over time consuming 0% control and 0.4% glucose fed OP50 *E. coli* measured from photos and quantified by pixel area per animal, Day 1 *C. elegans* consuming 0% control (n=27) and 0.4% glucose fed OP50 *E. coli* (n=30), **P ≤ 0.005; Day 6 *C. elegans* consuming 0% control (n=177) and 0.4% glucose fed OP50 *E. coli* (n=186), ***P ≤ 0.001; Day 12 *C. elegans* consuming 0% control (n=62) and 0.4% glucose fed OP50 *E. coli* (n=66), ***P ≤ 0.001. All Statistical analysis of *C. elegans* consuming either 0% control or 0.4% glucose fed OP50 *E. coli* compared at the same time point using an unpaired two-tailed t test with Graphpad Prism 8 software (ns = not significant P > 0.05, *P ≤ 0.05, **P ≤ 0.005, ***P ≤ 0.001). Data shown is a compilation from at least 3 biological replicates.

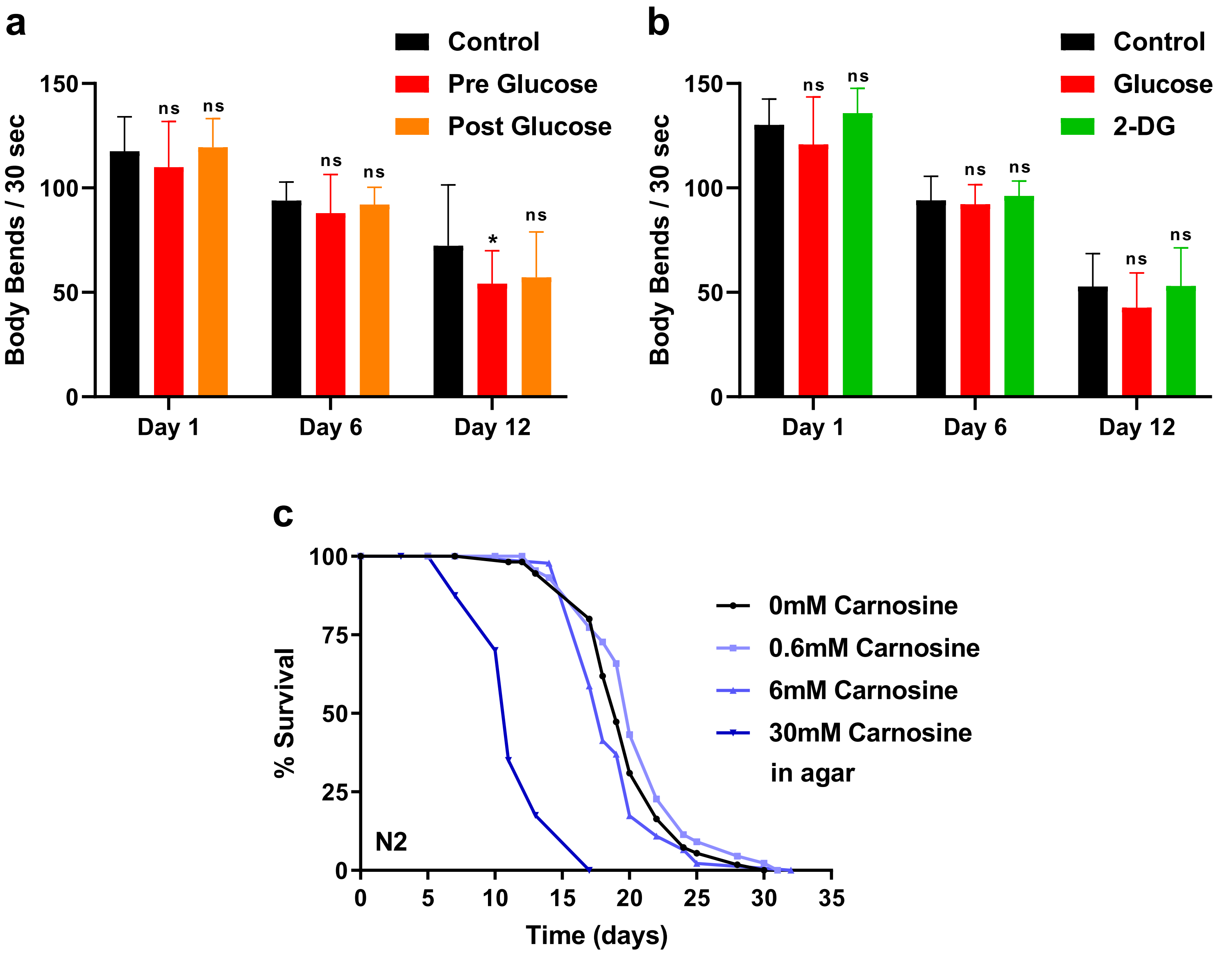

Supplementary Figure S2. Interfering with bacterial metabolism of glucose alters *C. elegans* physiology. (a) Healthspan – Movement in liquid/thrashing/swimming of wild type *C. elegans* consuming either 0% control (n=53), 0.4% glucose pre culture (n=54) or 0.4% glucose post culture (n=54) supplemented OP50 *E. coli* over time.(b) Healthspan – Movement in liquid/thrashing/swimming of wild type *C. elegans* consuming either 0% control (n=30), 0.4% glucose (n=32), or 0.4% 2-deoxy-glucose (2-DG; n=31) fed OP50 *E. coli* over time. (c) Lifespan of wild type *C. elegans* with 0, 0.6, 6, and 30mM Carnosine within the agar seeded with heat killed OP50 *E. coli*. The mean lifespans were as follows: Mean ± Standard Deviation (n= total number of animals assayed); wild type *C. elegans* with 0 mM Carnosine 18.7 ± 0.63 (n=60); wild type *C. elegans* with 0.6 mM Carnosine 19.4 ± 0.73^{ns} (n=54); wild type *C. elegans* with 6mM Carnosine 18.6 ± 0.56^{ns} (n=53); wild type *C. elegans* with 30mM Carnosine 10.1±1.2*** (n=60). All statistical analysis of histograms compares *C. elegans* consuming either 0% control or 0.4% glucose fed OP50 *E. coli* at the same time point using an unpaired two-tailed t test, and survival graph performed using a Log-rank Mantel-Cox test with Graphpad Prism 8 software (ns = not significant, *P ≤ 0.05, **P ≤ 0.005, ***P ≤ 0.001). Data shown is a compilation from at least 3 biological replicates.

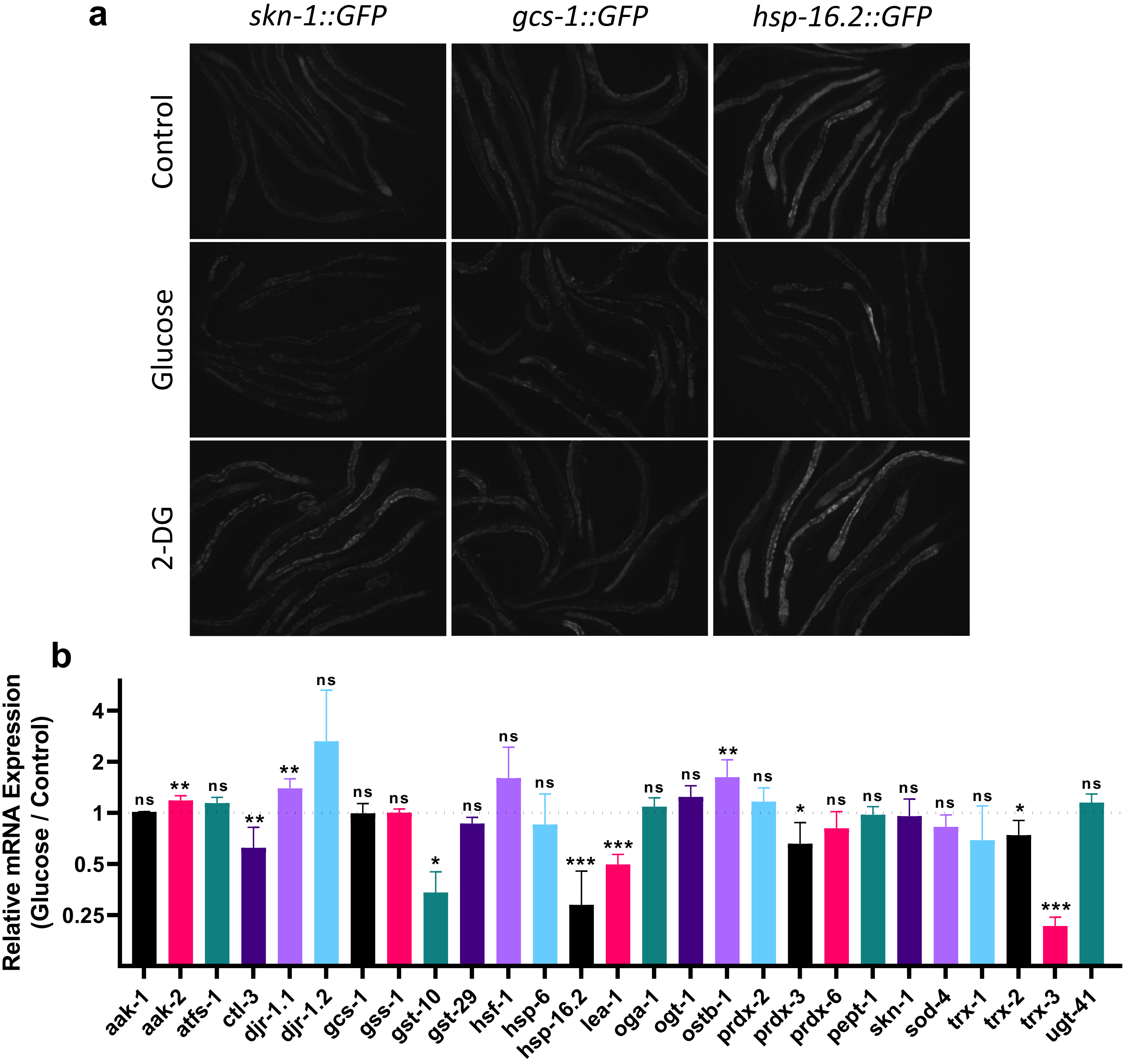

Supplementary Figure S3. *C. elegans* gene expression changes with bacterial metabolism of glucose. (a) Fluorescent imaging of transgenic *C. elegans* [*skn-1::gfp*], [*gcs-1::gfp*], or [*hsp-16.2::gfp*], after consuming either 0% control, 0.4% glucose, or 0.4% 2-deoxy-glucose (2-DG) fed OP50 *E. coli* for 6 days. (b) RTqPCR of wild type *C. elegans* consuming either 0% control or 0.4% glucose fed *E. coli* for 6 days displayed as relative expression of glucose fed *E. coli* compared to control. Statistical analysis performed using an unpaired two-tailed t test with Graphpad Prism 8 software (ns = not significant, *P ≤ 0.05, **P ≤ 0.005, ***P ≤ 0.001). All of the data was compiled from at least 3 biological replicates.

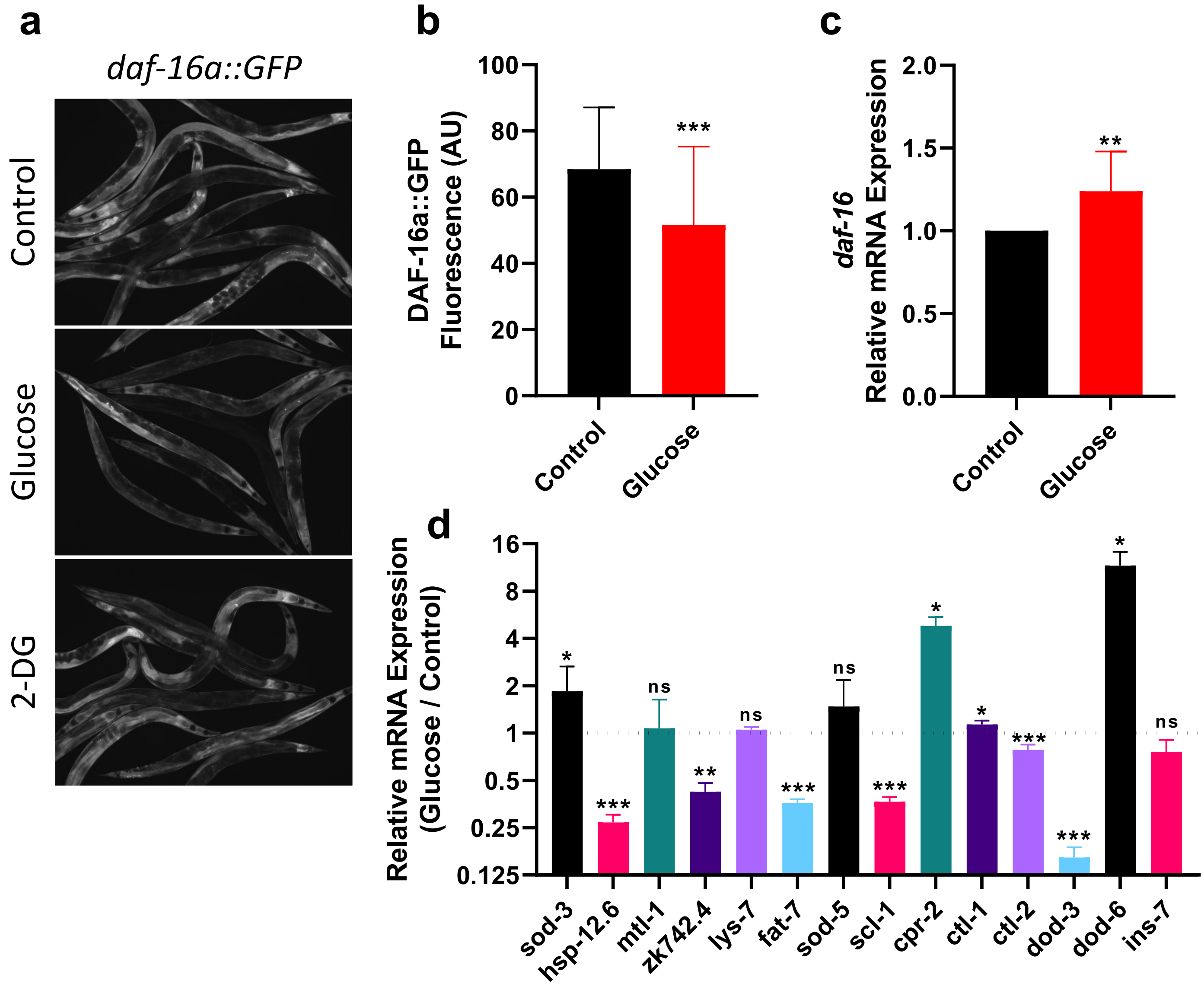

Supplementary Figure S4. *C. elegans daf-16* expression with bacterial metabolism of glucose. (a) Fluorescent imaging of transgenic *C. elegans* [*daf-16a::gfp*], after consuming either 0% control, 0.4% glucose, or 0.4% 2-deoxy-glucose (2-DG) fed OP50 *E. coli* for 6 days. (b) Fluorescence quantification of *daf-16a::gfp* animals in Figure S4a after consuming 0% control (n=40) and 0.4% glucose (n=40) fed OP50 *E. coli* for 6 days. (c) RTqPCR of *daf-16* mRNA in wild type *C. elegans* consuming either 0% control or 0.4% glucose fed *E. coli* for 6 days. (d) RTqPCR of *daf-16* target genes in wild type *C. elegans* consuming either 0% control or 0.4% glucose fed *E. coli* for 6 days, displayed as relative expression of glucose treatment compared to control. Statistical analysis performed using an unpaired two-tailed t test with Graphpad Prism 8 software (ns = not significant, *P ≤ 0.05, **P ≤ 0.005, ***P ≤ 0.001). Data shown was collected from at least 2 technical repeats.

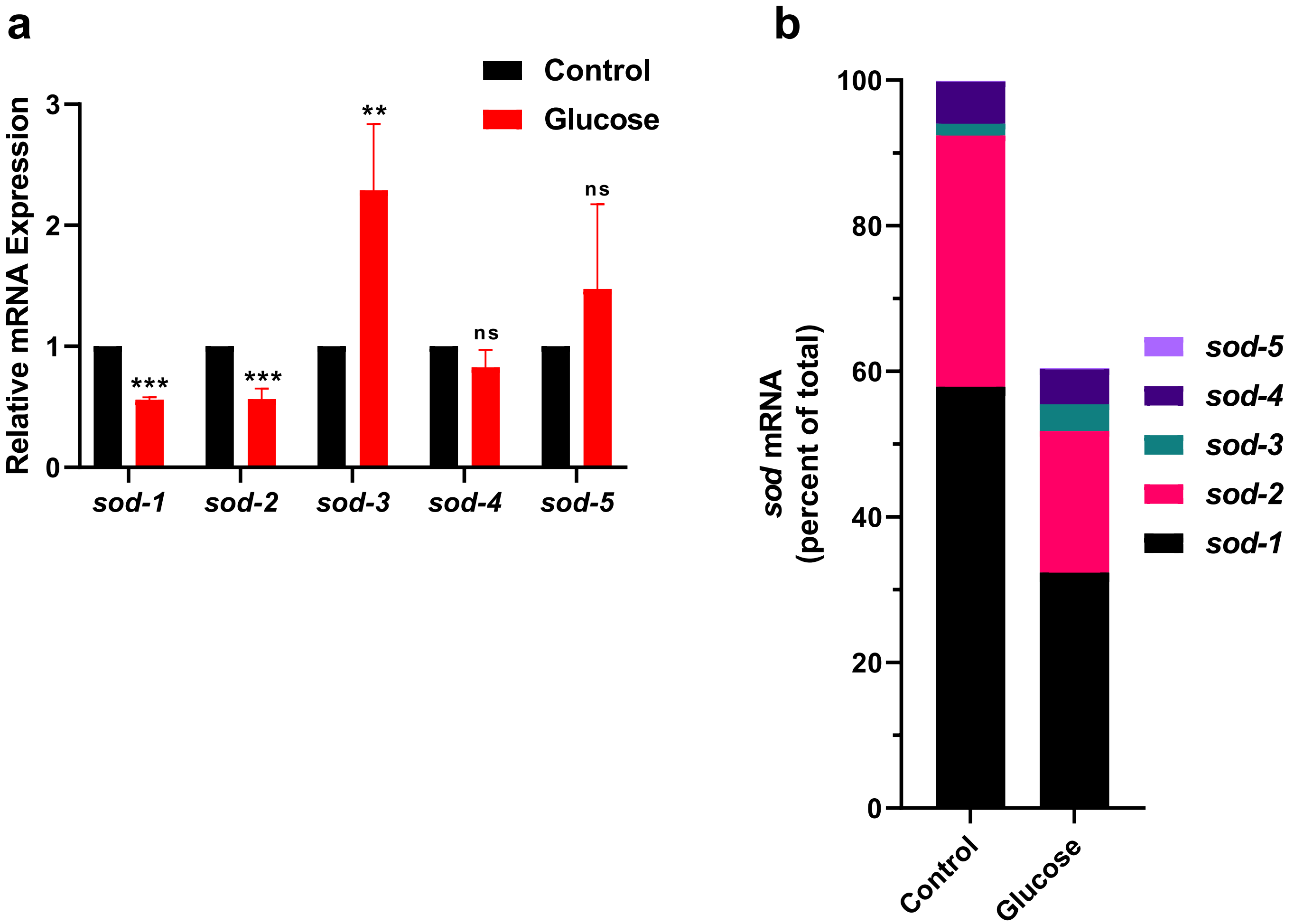

Supplementary Figure S5. *C. elegans sod* mRNA expression with bacterial metabolism of glucose. (a) RTqPCR experiments performed using wild type *C. elegans* consuming either 0% control and 0.4% glucose fed *E. coli* for 6 days, examining the 5 *sod* genes. (b) Estimated percent of total *sod* expression within *C. elegans* treated with glucose fed *E. coli* based off the preceding RTqPCR data and published RNA Seq expression ratio data (Dues et al 2017). Statistical analysis performed using an unpaired two-tailed t test with Graphpad Prism 8 software (ns = not significant, *P ≤ 0.05, **P ≤ 0.005, ***P ≤ 0.001), all experimental data collected from at least 3 biological replicates.

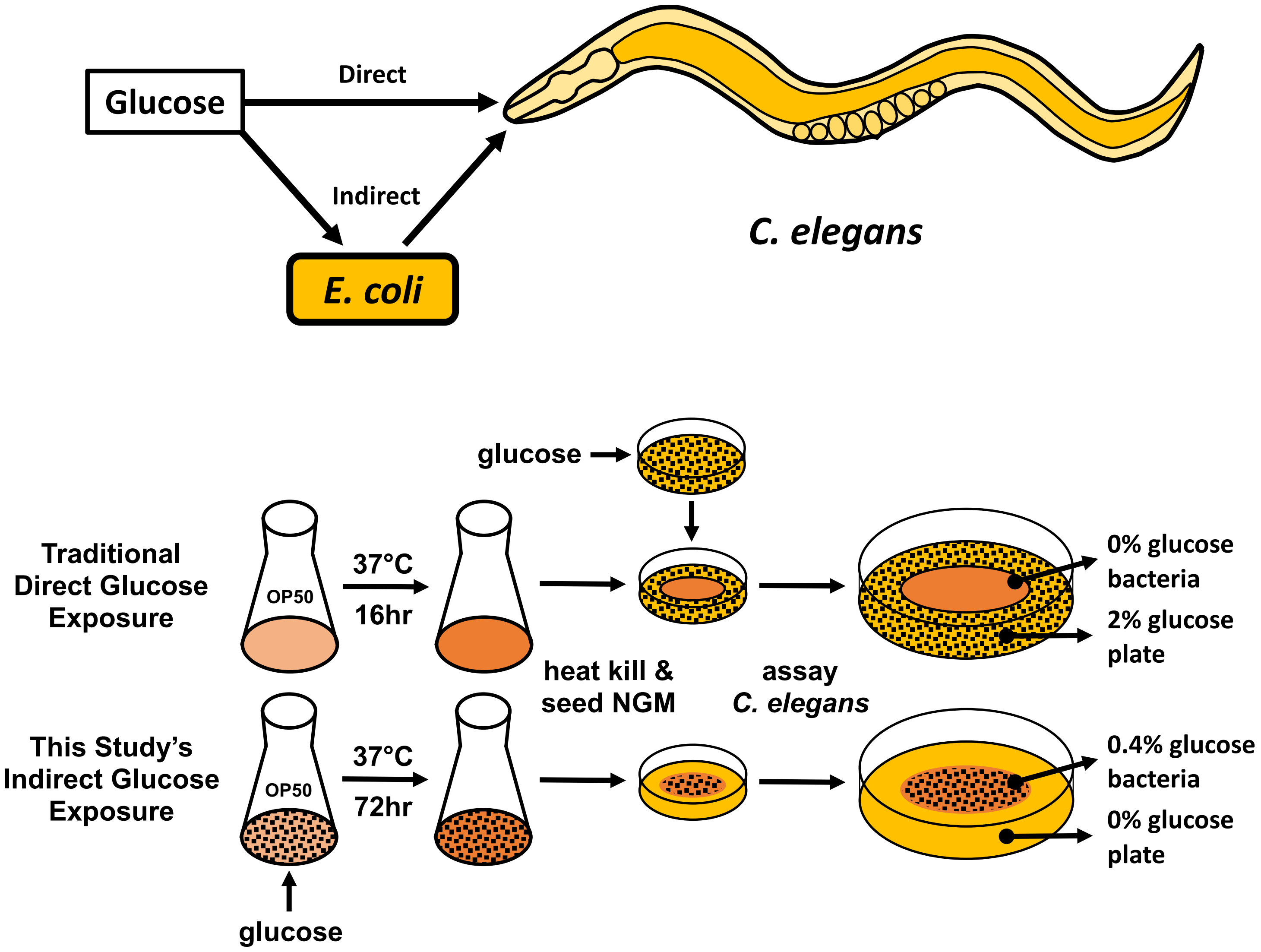

Supplementary Figure S6. Model illustration of direct and indirect glucose supplementation to *C. elegans*, and a diagram of the protocols that achieve either type of exposure method.

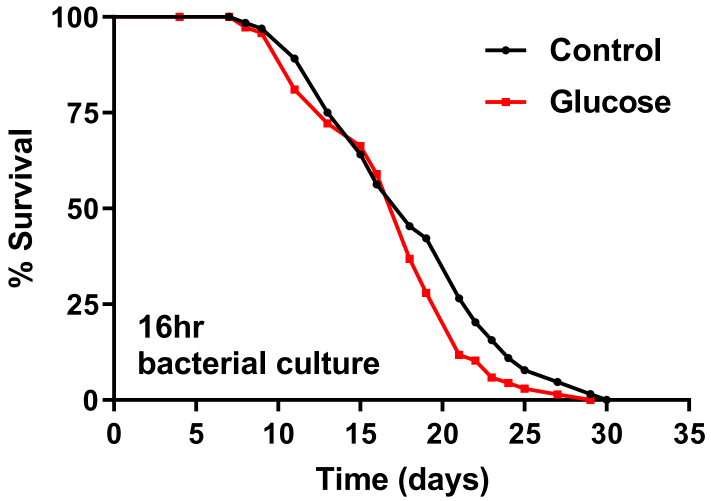

Supplementary Figure S7. Overnight (16hr) incubation of *E. coli* with glucose does not affect *C. elegans* lifespan. Lifespan of wild type *C. elegans* consuming either 0% control or 0.4% glucose fed OP50 *E. coli* for 16 hours. The mean lifespans were as follows: Mean ± Standard Deviation (n= total number of animals assayed) wild type *C. elegans* treated with 16 hour cultured control 18.3 ± 0.7 (n=67) and 0.4% glucose 17.1 ± 0.6^{ns} (n=77) fed OP50 *E. coli*. Statistical analysis performed using a Log-rank Mantel-Cox test with Graphpad Prism 8 software (ns = not significant). Data is a compilation of at least 3 biological replicates.

**Supplementary Table S1: Summary of pooled Lifespan statistics of *C. elegans* on all treatments**								
P values calculated against the appropriate control treatment using a Log-rank Mantel-Cox test. (*P ≤ 0.05, **P ≤ 0.005, ***P ≤ 0.001, ns = not significant)								
**Figure**	***C. elegans* Genotype**	***E. coli* Treatment**	**Mean Survival ± Std Dev (Days)**	**P value**	**P ***	**Animal Deaths**	**Censored Subjects**	**Repeats**
1D	Wild type (N2)	0% Control	21.2 ± 2.1	-		955	50	12
		0.4% Glucose	17.1 ± 2.9	0.0001	***	879	72	
2A	Wild type (N2)	0% Control	21.4 ± 1.6	-		361	14	4
		0.4% Glucose	17.9 ± 2.6	0.0001	***	345	9	
		0.4% Glucose Post Culture	20.6 ± 1.2	0.0199	*	354	17	
2B	Wild type (N2)	0% Control	21.4 ± 0.2	-		275	15	3
		0.4% Glucose	16.6 ± 2.3	0.0001	***	165	8	
		0.4% 2-DG	21.6 ± 1.2	0.0134	*	288	12	
2C	Wild type (N2)	0% Control	20.9 ± 1.9	-		238	16	3
		0.4% Glucose	15.4 ± 1.8	0.0001	***	207	28	
		50mM Carnosine	22.3 ± 1.3	0.0001	***	208	23	
		50mM Carnosine + 0.4% Glucose	19.4 ± 1.8	0.0001	***	244	26	
4B	Wild type (N2)	0% Control	19.1 ± 1.6	-		257	9	3
	gst-4(lp11)	0% Control	17.2 ± 1.2	0.0001	***	251	10	
	gst-4(lp10)	0% Control	17.3 ± 0.0	0.0001	***	90	2	
4C	Wild type (N2)	0% Control	20.3 ± 1.0	-		290	15	3
		0.4% Glucose	18.4 ± 0.5	0.0001	***	128	16	
	gst-4(lp11)	0% Control	19.0 ± 0.6	-		298	12	
		0.4% Glucose	18.9 ± 1.1	0.7508	ns	302	9	

**Supplementary Table S2: Individual Lifespan statistics of *C. elegans* on all treatments.**<br>* P values calculated against the appropriate control using a Log-rank Mantel-Cox test<br>(*P ≤ 0.05, **P ≤ 0.005, ***P ≤ 0.001, ns = not significant)								
**Figure**	***C. elegans* Genotype**	***E. coli* Treatment**	**Mean Survival (Days)**	**P value**	**P ***	**Animal Deaths**	**Censored Subjects**	**Repeat #**
1D	Wild type (N2)	0% Control	22.3	-		81	5	1
		0.4% Glucose	19.2	0.0031	**	68	1	
1D	Wild type (N2)	0% Control	20.0	-		60	2	2
		0.4% Glucose	18.3	0.0335	*	59	2	
1D	Wild type (N2)	0% Control	19.6	-		107	2	3
		0.4% Glucose	13.7	0.0001	***	102	2	
1D	Wild type (N2)	0% Control	23.5	-		113	5	4
		0.4% Glucose	20.7	0.0001	***	116	4	
1D	Wild type (N2)	0% Control	18.2	-		61	2	5
		0.4% Glucose	15.8	0.0736	ns	32	2	
1D	Wild type (N2)	0% Control	19.5	-		91	2	6
		0.4% Glucose	15.0	0.0001	***	109	3	
1D	Wild type (N2)	0% Control	23.4	-		70	1	7
		0.4% Glucose	15.2	0.0001	***	52	15	
1D	Wild type (N2)	0% Control	20.3	-		60	12	8
		0.4% Glucose	17.7	0.2699	ns	57	10	
1D	Wild type (N2)	0% Control	25.5	-		42	4	9
		0.4% Glucose	23.0	0.6152	ns	21	22	
1D	Wild type (N2)	0% Control	19.0	-		108	3	10
		0.4% Glucose	13.2	0.0001	***	98	3	

**Supplementary Table S2: Individual Lifespan statistics of *C. elegans* on all treatments.**<br>* P values calculated against the appropriate control using a Log-rank Mantel-Cox test.<br>(*P ≤ 0.05, **P ≤ 0.005, ***P ≤ 0.001, ns = not significant)								
**Figure**	***C. elegans* Genotype**	***E. coli* Treatment**	**Mean Survival (Days)**	**P value**	**P ***	**Animal Deaths**	**Censored Subjects**	**Repeat #**
1D	Wild type (N2)	0% Control	21.6	-		83	9	11
		0.4% Glucose	18.9	0.0001	***	89	5	
1D	Wild type (N2)	0% Control	21.4	-		79	3	12
		0.4% Glucose	14.4	0.0001	***	76	3	
2A	Wild type (N2)	0% Control	22.3	-		81	5	1
		0.4% Glucose	19.2	0.0031	**	68	1	
		0.4% Glucose Post Culture	21.2	0.254	ns	83	10	
2A	Wild type (N2)	0% Control	20.0	-		60	2	2
		0.4% Glucose	18.3	0.0335	*	59	2	
		0.4% Glucose Post Culture	19.5	0.3266	ns	62	2	
2A	Wild type (N2)	0% Control	19.6	-		107	2	3
		0.4% Glucose	13.7	0.0001	***	102	2	
		0.4% Glucose Post Culture	19.4	0.9425	ns	106	2	
2A	Wild type (N2)	0% Control	23.5	-		113	5	4
		0.4% Glucose	20.7	0.0001	***	116	4	
		0.4% Glucose Post Culture	22.3	0.0338	*	103	3	
2B	Wild type (N2)	0% Control	21.6	-		83	9	1
		0.4% Glucose	18.9	0.0001	***	89	5	
		0.4% 2-DG	19.9	0.0015	**	83	6	

**Supplementary Table S2: Individual Lifespan statistics of *C. elegans* on all treatments.**<br>* P values calculated against the appropriate control using a Log-rank Mantel-Cox test.<br>(*P ≤ 0.05, **P ≤ 0.005, ***P ≤ 0.001, ns = not significant)								
**Figure**	***C. elegans* Genotype**	***E. coli* Treatment**	**Mean Survival (Days)**	**P value**	**P ***	**Animal Deaths**	**Censored Subjects**	**Repeat #**
2B	Wild type (N2)	0% Control	21.4	-		79	3	2
		0.4% Glucose	14.4	0.0001	***	76	3	
		0.4% 2-DG	22.4	0.0175	*	94	3	
2B	Wild type (N2)	0% Control	21.1	-		113	3	3
		0.4% Glucose				0	0	
		0.4% 2-DG	22.3	0.0041	**	110	3	
2C	Wild type (N2)	0% Control	23.4	-		70	1	1
		0.4% Glucose	15.2	0.0001	***	52	15	
		50mM Carnosine	22.0	0.1111	ns	65	3	
		50mM Carnosine + 0.4% Glucose	19.1	0.0001	***	69	1	
2C	Wild type (N2)	0% Control	19.0	-		108	3	2
		0.4% Glucose	13.2	0.0001	***	98	3	
		50mM Carnosine	20.9	0.0032	**	87	3	
		50mM Carnosine + 0.4% Glucose	17.4	0.002	**	125	3	
2C	Wild type (N2)	0% Control	20.3	-		60	12	3
		0.4% Glucose	17.7	0.2699	ns	57	10	
		50mM Carnosine	24.0	0.0001	***	56	17	
		50mM Carnosine + 0.4% Glucose	21.8	0.0256	*	49	22	
4B	Wild type (N2)	0% Control	16.9	-		67	3	1
	gst-4(lp11)	0% Control	16.1	0.0557	ns	61	5	
	gst-4(lp10)	0% Control				0	0	

**Supplementary Table S2: Individual Lifespan statistics of *C. elegans* on all treatments.**<br>* P values calculated against the appropriate control using a Log-rank Mantel-Cox test.<br>(*P ≤ 0.05, **P ≤ 0.005, ***P ≤ 0.001, ns = not significant)								
**Figure**	***C. elegans* Genotype**	***E. coli* Treatment**	**Mean Survival (Days)**	**P value**	**P ***	**Animal Deaths**	**Censored Subjects**	**Repeat #**
4B	Wild type (N2)	0% Control	20.0	-		86	3	2
	gst-4(lp11)	0% Control	16.8	0.0001	***	86	3	
	gst-4(lp10)	0% Control	17.3	0.0001	***	90	2	
4B	Wild type (N2)	0% Control	20.3	-		104	3	3
	gst-4(lp11)	0% Control	18.8	0.0011	**	104	3	
	gst-4(lp10)	0% Control				0	0	
4C	Wild type (N2)	0% Control	21.6	-		83	9	1
		0.4% Glucose	18.9	0.0001	***	89	5	
	gst-4(lp11)	0% Control	19.8	-		85	6	
		0.4% Glucose	20.2	0.0894	ns	88	3	
4C	Wild type (N2)	0% Control	19.1	-		103	3	2
		0.4% Glucose				0	0	
	gst-4(lp11)	0% Control	18.4	-		109	3	
		0.4% Glucose	17.6	0.0638	ns	112	3	
4C	Wild type (N2)	0% Control	20.3	-		104	3	3
		0.4% Glucose	17.9	0.0001	***	39	11	
	gst-4(lp11)	0% Control	18.8	-		104	3	
		0.4% Glucose	18.9	0.7574	ns	102	3	

Supplementary Table S3: RT-qPCR, PCR, and CRISPR primer sequences			
**Gene**	**Use**	**Sequence**	**Source**
act-1	RT-qPCR	CTCTTGCCCCATCAACCATG	Kwon et al 2010
act-1	RT-qPCR	CTTGCTTGGAGATCCACATC	Kwon et al 2010
act-1	RT-qPCR	GGAGTCATGGTCGGTATGG	GETprime Ensembl release 81
act-1	RT-qPCR	CTTGAGGGTAAGGATACCTCTC	GETprime Ensembl release 81
atfs-1	RT-qPCR	TTGGAGATAATATGGGCTCCC	GETprime Ensembl release 81
atfs-1	RT-qPCR	CTATTCGGGAAGTTCCCGT	GETprime Ensembl release 81
cpr-2	RT-qPCR	CTGCGTAAACCTTCAAACTC	Kwon et al 2010
cpr-2	RT-qPCR	ATGCGGAGTTACCATAGTTC	Kwon et al 2010
ctl-1	RT-qPCR	CCAATGCTCATGCAAGATGT	Schaar et al 2015
ctl-1	RT-qPCR	TTGCGTCACGAATGAAGAAG	Schaar et al 2015
ctl-2	RT-qPCR	ACGTCCTTGGAGCATCTTGT	Schaar et al 2015
ctl-2	RT-qPCR	GCAAGATGGTGCTGAACAGA	Schaar et al 2015
ctl-3	RT-qPCR	CTTCCCCACATGGTCAATCT	Schaar et al 2015
ctl-3	RT-qPCR	TGTCCTGCATTAGCATTGGA	Schaar et al 2015
daf-16	RT-qPCR	TCCATCATCTTTCCGTCCC	GETprime Ensembl release 81
daf-16	RT-qPCR	CTTCCAATAGCTGGAGAAACAC	GETprime Ensembl release 81
djr-1.1	RT-qPCR	TTGAGCCATGGAGTCAAGG	GETprime Ensembl release 81
djr-1.1	RT-qPCR	AGTACTTGTAGCCTCCTTTCTC	GETprime Ensembl release 81
djr-1.2	RT-qPCR	CTGAACCTGTCAAATGTGCC	GETprime Ensembl release 81
djr-1.2	RT-qPCR	TGTCGAATGTCTTGTTCTTCAC	GETprime Ensembl release 81
dod-3	RT-qPCR	GTGCATATTGTGGAGCTGC	GETprime Ensembl release 81
dod-3	RT-qPCR	ATAGTTGGTCGGACGTTGG	GETprime Ensembl release 81
dod-6	RT-qPCR	GATGCTGAAGATGGTCACTG	GETprime Ensembl release 81
dod-6	RT-qPCR	AAGTCTTTGGGCACTTGGA	GETprime Ensembl release 81
fat-7	RT-qPCR	ATAGTGTGGCGTAACGTGGC	Kwon et al 2010
fat-7	RT-qPCR	TAGAGAGCAAATGAGAAGACG	Kwon et al 2010
gcs-1	RT-qPCR	CCAATCGATTCCTTTGGAGAC	GETprime Ensembl release 81
gcs-1	RT-qPCR	CGATGAGACCTCCGTAAGG	GETprime Ensembl release 81
gss-1	RT-qPCR	AAGGAAGGATGCACCTGAG	GETprime Ensembl release 81
gss-1	RT-qPCR	GCTACTCCACCCATAGCTG	GETprime Ensembl release 81
gst-10	RT-qPCR	CTTCACTATTCGAGGATTCGG	GETprime Ensembl release 81
gst-10	RT-qPCR	TCGAACCGAATGTCTTCGA	GETprime Ensembl release 81
gst-29	RT-qPCR	ATGGAGATGGTACATGGGA	GETprime Ensembl release 81
gst-29	RT-qPCR	TAGAACTGGAACTTGGCCA	GETprime Ensembl release 81
gst-4	RT-qPCR	GCTGAAGCCAACGACTCCAT	Park, S-K. et al 2009
gst-4	RT-qPCR	GACCGAATTGTTCTCCATCGA	Park, S-K. et al 2009
gst-4	CRISPR	AAATACAATAGCTTATAGTT	http://crispor.tefor.net
gst-4	CRISPR	TCGTTGGAGACTCATTGACT	http://crispor.tefor.net
gst-4	PCR	TGATGCCAGACGATGACATTAC	IDTdna PrimerQuest tool
gst-4	PCR	TCTCTGGGAGACGTGATAGG	IDTdna PrimerQuest tool
gst-4	PCR	CAAATTTCCAGCGACTCCATTT	IDTdna PrimerQuest tool
gst-4	PCR	ATGATCAGCGTCACTTCCATAG	IDTdna PrimerQuest tool

Supplementary Table S3: RT-qPCR, PCR, and CRISPR primer sequences

Gene	Use	Sequence	Source
hsf-1	RT-qPCR	TCAGACAGTTGAATATGTACGG	GETprime Ensembl release 81
hsf-1	RT-qPCR	CCTGATCTGATTCTGTTCGAG	GETprime Ensembl release 81
hsp-12.6	RT-qPCR	TGGAGTTGTCAATGTCCTCG	Kwon et al 2010
hsp-12.6	RT-qPCR	GACTTCAATCTCTTTTGGGAGG	Kwon et al 2010
hsp-16.2	RT-qPCR	TGGTGCAGTTGCTTCGAATC	Park, S-K. et al 2009
hsp-16.2	RT-qPCR	TTGAACCGCTTCTTTCTTTGG	Park, S-K. et al 2009
hsp-6	RT-qPCR	GAAGATACGAAGACCCAGAGGTTC	Pellegrino MW et al 2014
hsp-6	RT-qPCR	CAACCTGAGATGGGGAATACACT	Pellegrino MW et al 2014
ins-7	RT-qPCR	AGAACCAGAAGAGTCCCTGA	Kwon et al 2010
ins-7	RT-qPCR	ATATTAACTTCAGTATTCGATTCG	Kwon et al 2010
lys-7	RT-qPCR	GCCGTCAAACTTGGCATCTT	Kwon et al 2010
lys-7	RT-qPCR	GGGTTGTATGCACGAACGAA	Kwon et al 2010
mtl-1	RT-qPCR	AATCATGGCTTGCAAGTGTG	GETprime Ensembl release 81
mtl-1	RT-qPCR	TTCACATTTGTCTCCGCAC	GETprime Ensembl release 81
oga-1	RT-qPCR	ACATTATGTGGACAGGACCTC	GETprime Ensembl release 81
oga-1	RT-qPCR	GACGCATTACACTTCCCAC	GETprime Ensembl release 81
ogt-1	RT-qPCR	TGATCATGACAGGACAAATGAC	GETprime Ensembl release 81
ogt-1	RT-qPCR	GATGCATTTGAGACTGTCCG	GETprime Ensembl release 81
ostb-1	RT-qPCR	ATGATGTGCAACAGGTGTC	GETprime Ensembl release 81
ostb-1	RT-qPCR	CGTAGTATGGATATGCGGAG	GETprime Ensembl release 81
pept-1	RT-qPCR	TGCAACACTGGTATTTATGGG	GETprime Ensembl release 81
pept-1	RT-qPCR	CGAGATACTTCTCCGAACAC	GETprime Ensembl release 81
prdx-2	RT-qPCR	GGCCTGAACAAGACGAAGAG	Schaar et al 2015
prdx-2	RT-qPCR	GTGTGCCCAACCGAGATTAT	Schaar et al 2015
prdx-3	RT-qPCR	CAGCTCCAAGAGAACGGAAC	Schaar et al 2015
prdx-3	RT-qPCR	CCAGCTTTCAAAGGAACTGC	Schaar et al 2015
prdx-6	RT-qPCR	TGCTGTGATGCTTTTTGGAC	Schaar et al 2015
prdx-6	RT-qPCR	TCAGAGATGGTTGAGCGATG	Schaar et al 2015
scl-1	RT-qPCR	CAATCAAGCATTGTGGATGC	Kwon et al 2010
scl-1	RT-qPCR	GGAATCCACGACCATTTTCC	Kwon et al 2010
skn-1	RT-qPCR	GCAACAGCTACTCAATCGT	GETprime Ensembl release 81
skn-1	RT-qPCR	TGATGACGAATCAGTAGTGC	GETprime Ensembl release 81
sod-1	RT-qPCR	ACTGTCGTTGGCCGATCTATG	Schaar et al 2015
sod-1	RT-qPCR	CGCCGAGGTCGTCTTGTC	Schaar et al 2015
sod-2	RT-qPCR	CAAAGGACGGAGGAGAACCA	Schaar et al 2015
sod-2	RT-qPCR	CGCTCTTAATTGCGGTGAGC	Schaar et al 2015
sod-3	RT-qPCR	AAAGGAGCTGATGGACACTATTAAGC	Schaar et al 2015
sod-3	RT-qPCR	AAGTTATCCAGGGAACCGAAGTC	Schaar et al 2015
sod-4	RT-qPCR	TTGAAGCCGCTTCCGAAG	Schaar et al 2015
sod-4	RT-qPCR	CAGCTTTGAAAATGTAGGCACGT	Schaar et al 2015
sod-5	RT-qPCR	CTGTTATCGGACGTTCCATGGT	Schaar et al 2015
sod-5	RT-qPCR	GGCCAAGATCATCTCGATCG	Schaar et al 2015

Supplementary Table S3: RT-qPCR, PCR, and CRISPR primer sequences			
**Gene**	**Use**	**Sequence**	**Source**
trx-1	RT-qPCR	CGTCAACATCCGGAGAAGAT	Schaar et al 2015
trx-1	RT-qPCR	TGCTCCAACACTTTTTGACG	Schaar et al 2015
trx-2	RT-qPCR	GTTGATTTCCACGCAGAATG	Schaar et al 2015
trx-2	RT-qPCR	TGGCGAGAAGAACACTTCCT	Schaar et al 2015
trx-3	RT-qPCR	TGGTAGATGCAGGAGAGCAC	Schaar et al 2015
trx-3	RT-qPCR	TCCAACTGGAATCGCATGTA	Schaar et al 2015
ugt-41	RT-qPCR	CTCTTCTAGCTGATTCCCGT	GETprime Ensembl release 81
ugt-41	RT-qPCR	TTCCGAGGTAGCTCAACTC	GETprime Ensembl release 81
ZK742.4	RT-qPCR	ACTGGAGGTTGGCAAACTGC	Kwon et al 2010
ZK742.4	RT-qPCR	TACGAGGCAAATCTGGCTCA	Kwon et al 2010

References

1. Kwon, E. S., Narasimhan, S. D., Yen, K. & Tissenbaum, H. A. A new DAF-16 isoform regulates longevity. *Nature* **466**, 498-502, doi:10.1038/nature09184 (2010).
2. Schaar, C. E. *et al.* Mitochondrial and cytoplasmic ROS have opposing effects on lifespan. *PLoS Genet* **11**, e1004972, doi:10.1371/journal.pgen.1004972 (2015).
3. Park, S. K., Tedesco, P. M. & Johnson, T. E. Oxidative stress and longevity in Caenorhabditis elegans as mediated by SKN-1. *Aging Cell* **8**, 258-269, doi:ACE473 [pii]10.1111/j.1474-9726.2009.00473.x (2009).
4. Pellegrino, M. W. *et al.* Mitochondrial UPR-regulated innate immunity provides resistance to pathogen infection. *Nature* **516**, 414-417, doi:10.1038/nature13818 (2014).
